# Rapamycin directly activates lysosomal mucolipin TRP channels independent of mTOR

**DOI:** 10.1371/journal.pbio.3000252

**Published:** 2019-05-21

**Authors:** Xiaoli Zhang, Wei Chen, Qiong Gao, Junsheng Yang, Xueni Yan, Han Zhao, Lin Su, Meimei Yang, Chenlang Gao, Yao Yao, Ken Inoki, Dan Li, Rong Shao, Shiyi Wang, Nirakar Sahoo, Fumitaka Kudo, Tadashi Eguchi, Benfang Ruan, Haoxing Xu

**Affiliations:** 1 Department of Molecular, Cellular, and Developmental Biology, University of Michigan, Ann Arbor, Michigan, United States of America; 2 Collaborative Innovation Center of Yangtze River Delta Region Green Pharmaceuticals, College of Pharmaceutical Sciences, Zhejiang University of Technology, Hangzhou, China; 3 Department of Neurology, The Fourth Hospital of Harbin Medical University, Harbin, China; 4 Department of Integrative and Molecular Physiology and Internal Medicine, Life Sciences Institute, University of Michigan, Ann Arbor, Michigan, United States of America; 5 Department of Chemistry, Tokyo Institute of Technology, Ookayama, Meguro-ku, Tokyo, Japan; Institute of Basic Medical Sciences, NORWAY

## Abstract

Rapamycin (Rap) and its derivatives, called rapalogs, are being explored in clinical trials targeting cancer and neurodegeneration. The underlying mechanisms of Rap actions, however, are not well understood. Mechanistic target of rapamycin (mTOR), a lysosome-localized protein kinase that acts as a critical regulator of cellular growth, is believed to mediate most Rap actions. Here, we identified mucolipin 1 (transient receptor potential channel mucolipin 1 [TRPML1], also known as MCOLN1), the principle Ca^2+^ release channel in the lysosome, as another direct target of Rap. Patch-clamping of isolated lysosomal membranes showed that micromolar concentrations of Rap and some rapalogs activated lysosomal TRPML1 directly and specifically. Pharmacological inhibition or genetic inactivation of mTOR failed to mimic the Rap effect. In vitro binding assays revealed that Rap bound directly to purified TRPML1 proteins with a micromolar affinity. In both healthy and disease human fibroblasts, Rap and rapalogs induced autophagic flux via nuclear translocation of transcription factor EB (TFEB). However, such effects were abolished in TRPML1-deficient cells or by TRPML1 inhibitors. Hence, Rap and rapalogs promote autophagy via a TRPML1-dependent mechanism. Given the demonstrated roles of TRPML1 and TFEB in cellular clearance, we propose that lysosomal TRPML1 may contribute a significant portion to the in vivo neuroprotective and anti-aging effects of Rap via an augmentation of autophagy and lysosomal biogenesis.

## Abstract 2

The protein kinase mTOR is believed to mediate most of the effects of the drug rapamycin, but this study identifies MCOLN1, a lysosome-localized calcium channel that plays essential roles in autophagy and lysosome function, as a novel molecular target of rapamycin.

## Introduction

Rapamycin (Rap) is a natural macrocyclic compound that was initially isolated from *Streptomyces hygroscopicus* as an antifungal agent [1]. Because Rap was shown to have robust immunosuppressive and antiproliferative efficacy [2], Rap derivatives (rapalogs; see S1 Fig) with improved pharmacokinetic properties have been developed in the industry, including temsirolimus (Tem), everolimus (Eve), deforolimus (Defo), zotarolimus (Zota), WYE-592, and ILS-920 [3, 4]. Since 1999, Rap (brand name Sirolimus) and several rapalogs have been approved by the United States Food and Drug Administration for clinical trials testing their ability to target cancer cells and to alleviate metabolic and neurodegenerative diseases [3, 4]. More recently, Rap was also shown to extend life span across diverse organisms ranging from flies to mammals [4, 5]. Hence, elucidating the molecular mechanisms of Rap bioactivities is of great value for both basic and clinical research.

The first identified target protein of Rap was discovered in yeast and named target of rapamycin (TOR) [6, 7]. TOR, now renamed mechanistic target of rapamycin (mTOR), is a serine and/or threonine kinase that is highly conserved in eukaryotes [6, 7]. Although multiple cellular locations have been reported, there is now a consensus that mTOR is localized predominantly on the membranes of lysosomes under nutrient-rich conditions [8]. In response to environmental changes, such as nutrient availability, mTOR kinase activity is switched on and off through the formation of alternate protein complexes—mTOR complex 1 (mTORC1) and mTORC2—and through association with and dissociation from lysosomal membranes. Known mTOR substrates include, but are not limited to, UNC-5–like autophagy activating kinase (ULK1; also known as autophagy-related protein 1 homolog), p70 ribosomal protein S6 kinase (S6K), 4E binding protein 1 (4E-BP1), and transcription factor EB (TFEB) [9]. Rap acts as a high-affinity (nM range) allosteric inhibitor of mTORC1 (hereafter referred to as mTOR) that blocks mTOR substrate recruitment by binding to the FK506 binding protein (FKBP) and the rapamycin binding (FRB) domain of mTOR, forming a ternary FKBP12-Rap-mTOR complex [3, 4].

Both the anticancer and immunosuppressive effects of Rap are likely due to its inhibition of cell proliferation via mTOR, which integrates a number of signaling pathways in the cell and has thus emerged as a major regulator of cellular proliferation and growth [7]. However, mTOR inhibition also induces autophagy, a lysosome-dependent cellular survival mechanism that supplies recycled nutrients by degrading obsolete cellular components [10]. Defective autophagy may hasten aging and enable the pathogenesis of numerous diseases, including cancer and neurodegenerative diseases [4]. Hence, autophagy induction caused by mTOR inhibition may also explain many of the reported effects of Rap, especially neuroprotection and anti-aging effects [2, 11].

The basic autophagic process consists of autophagosome formation, autophagosome–lysosome fusion, and lysosomal degradation [12]. Nutrient insufficiency is a potent inducer of autophagy, in which the loss of nutrients (e.g., amino acids) causes mTOR inhibition. Subsequently, dephosphorylation of ULK1, a major mTOR target, primes phagophore initiation [12]. Rap can mimic the effect of starvation on ULK1-mediated autophagy induction [12]. Although all rapalogs inhibit mTOR potently, their clinical efficacies vary [13]. Rapalogs with relatively low mTOR binding affinities (e.g., WYE-592 and ILS-920) exhibit neuroprotective effects at least as potent as that of their counterparts with higher mTOR binding affinities [3]. Furthermore, although mTOR is inhibited much more potently by its catalytic inhibitors (e.g. Torin-1), in vivo beneficial effects have not been observed for these potent inhibitors [14]. Hence, Rap may have other targets besides mTOR in the autophagy pathway.

Sustained autophagy requires lysosome activation, reformation, and biogenesis [12, 15, 16]. Under conditions when lysosome function is compromised, such as in neurodegenerative diseases and lysosome storage diseases (LSDs), it is unlikely that an increase in autophagosome formation alone could produce beneficial effects related to cellular clearance. Nutrient starvation, a physiological inducer of autophagy, promotes both autophagosome formation and lysosome biogenesis. Upon starvation-induced mTOR inhibition, TFEB, a key regulator of autophagy and lysosome biogenesis [17], undergoes rapid activation via dephosphorylation and cytosol-to-nucleus translocation [17–20]. Starvation may also activate mucolipin 1 (MCOLN1; also known as transient receptor potential channel mucolipin 1 [TRPML1]), a lysosomal Ca^2+^ channel required for TFEB activation via the Ca^2+^-dependent phosphatase calcineurin [21, 22]. Activation of TFEB, in turn, up-regulates TRPML1 expression [23]. Therefore, TRPML1 and TFEB may constitute a positive-feedback loop that boosts lysosomal biogenesis and autophagy under lysosomal stress conditions. Indeed, up-regulation of either TFEB or TRPML1 has been reported to benefit several LSDs, including Pompe disease and Niemann-Pick type C (NPC) disease, as well as common neurodegenerative diseases, including Alzheimer disease [15, 17, 24, 25].

In the present study, we found that the TRPML1-TFEB-autophagy pathway is directly activated by Rap and some rapalogs. Employing biomolecular interaction assays and whole-endolysosome electrophysiology, we demonstrated that Rap bound directly to TRPML1 and specifically activated TRPML1 independent of mTOR.

## Results

### Direct activation of lysosomal TRPML1 channels by Rap

**Fig 1:**
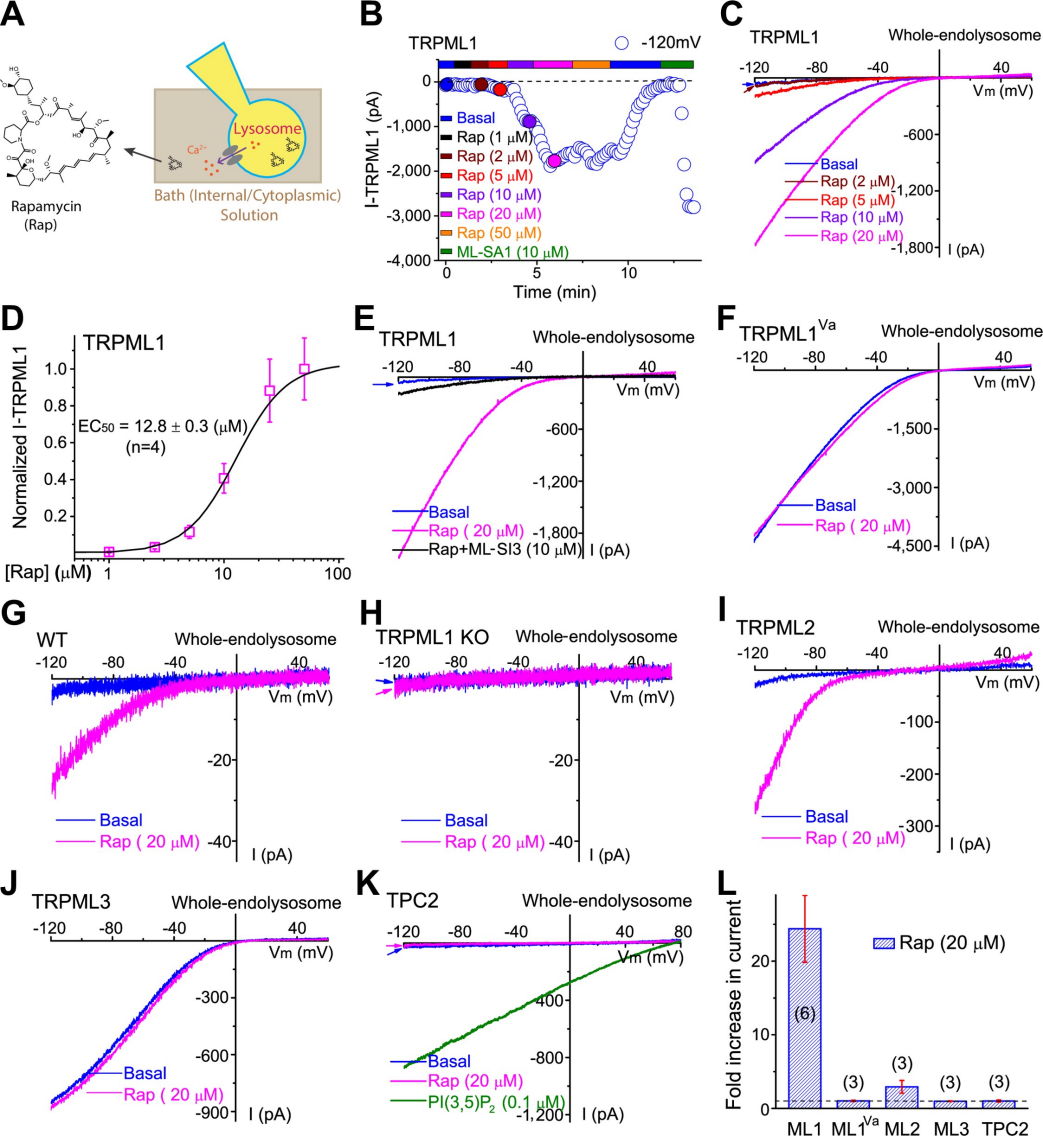
Direct activation of lysosomal TRPML1 channels by Rap. (A) Whole-endolysosome recording configuration. Pipette (luminal) solution was standard Tyrode’s solution with the pH adjusted to 4.6 to mimic the lysosomal lumen. Bath (internal) solution was a K^+^-based solution (140 mM K^+^-gluconate). Inward currents indicate cations flowing out. (B) Representative time course of whole-endolysosome TRPML1-mediated currents (*I*_TRPML1_, open circles, at −120 mV) activated by bath application of Rap (in μM: 1, 2, 5, 10, 20, 50). *I*_TRPML1_ was recorded from an enlarged vacuole isolated from EGFP-TRPML1–transfected COS1 cells. Currents were elicited by repeated voltage ramps (−120 to +120 mV; 200 ms) with a 4-s interstep interval. (C) Representative *I*_TRPML1_ by 2 μM, 5 μM, 10 μM, and 20 μM Rap (time points as in panel B). Partial voltage protocol is shown (holding potential, 0 mV). (D) Dose-dependent activation of TRPML1 by Rap. (E) Rap-evoked *I*_TRPML1_ was blocked by coapplication of ML-SI3, a synthetic inhibitor of TRPML1. (F) Constitutively active *I*_TRPML1-Va_ was not affected by Rap. (G) Rap evoked endogenous *I*_TRPML1_ in *WT* parietal cells. (H) No Rap-induced *I*_TRPML1_ was detected in *TRPML1* KO parietal cells. (I) Whole-endolysosome *I*_TRPML2_ was activated by Rap in mCherry-TRPML2–transfected COS1 cells. (J) Rap did not activate *I*_TRPML3_. (K) Rap did not produce activation of whole-endolysosome *I*_TPC2_ in EGFP-TPC2–transfected COS1 cells. (L) Summary of Rap effects on TRPML1, 2, and 3, and TPC2. Data are presented as mean ± SEM. Dashed line indicates 1 (no change in current). Only representative data are shown in (E–K). The individual data underlying (D) and (L) can be found in S1 Data. COS1, CV-1 in Origin Simian-1; EC50, half maximal effective concentration; EGFP, enhanced green fluorescent protein; KO, knockout; mCherry, a monomeric red fluorescent protein; ML, TRPML; ML-SA1, TRPML1 synthetic agonist 1; ML-SI3, TRPML1 synthetic inhibitor 3; Rap, rapamycin; TPC2, two-pore channel 2; TRPML1, transient receptor potential channel mucolipin 1; WT, wild type.

Given TRPML1’s proposed roles in lysosomal membrane trafficking and cellular clearance [24], we used Ca^2+^ imaging and electrophysiological assays to screen for potential TRPML1 modulators from a list of natural products that are known to affect lysosome function or autophagy. Whole-endolysosome recordings were performed in vacuoles that had been enlarged with vacuolin-1 and isolated manually from enhanced green fluorescent protein (EGFP)-TRPML1–transfected CV-1 in Origin Simian-1 (COS1) cells [26] (Fig 1A). We found that Rap induced robust activation of whole-endolysosomal TRPML1 current (*I*_TRPML1_; Fig 1B and 1C). The activation had a half-maximal effective concentration of 12.8 ± 1.0 μM (*n* = 4 patches; Fig 1C and 1D), demonstrating potency less than that of the endogenous agonist phosphatidylinositol 3,5-bisphosphate (PI(3,5)P_2_) but comparable to that of the TRPML1 synthetic agonist 1 (ML-SA1) [25]. Like the currents evoked by the known agonists, Rap-evoked *I*_TRPML1_ was inhibited by TRPML1 synthetic inhibitors (ML-SIs), e.g., ML-SI3 [22] (also see Fig 1E). On the other hand, Rap failed to affect the constitutively active mutant TRPML1 channels (TRPML1^Va^; Fig 1F). Furthermore, endogenous *I*_TRPML1_ was activated by Rap in wild-type (WT) but not in *TRPML1* knockout (KO) parietal cells (Fig 1G and 1H). In contrast, whole-endolysosome *I*_TRPML3_ and *I*_TPC2_ (two-pore channel 2) were not affected by Rap (Fig 1J–1L); mild but significant activation was observed in TRPML2-expressing cells (Fig 1I and 1L). Rap also had synergistic effects on *I*_TRPML1_ with PI(3,5)P_2_, the endogenous agonist of TRPML1 [27] (S1E Fig). These results suggest that Rap is a specific and robust activator of TRPML1.

### TRPML1 activation by Rap and rapalogs is independent of mTOR

**Fig 2:**
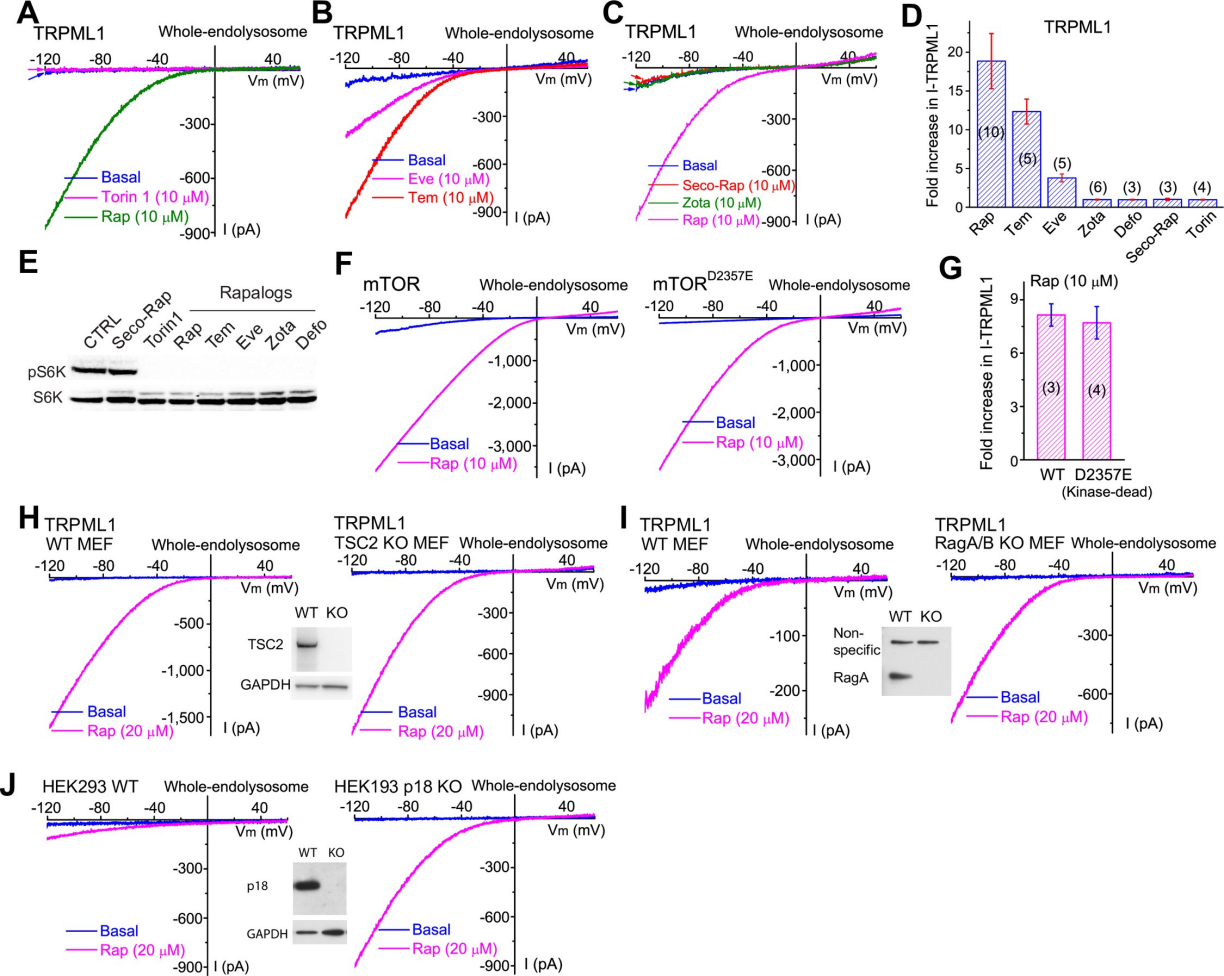
Rap and rapalogs activate TRPML1 in an mTOR-independent manner. (A) Effect of Torin-1 (10 μM), a potent ATP-competitive mTOR inhibitor, on *I*_TRPML1_. (B) Tem (10 μM) and Eve (10 μM) stimulation of *I*_TRPML1_. (C) No effects of Defo (10 μM), Zota (10 μM), and Seco-Rap (a Rap metabolite, 10 μM) on *I*_TRPML1_ measured at −120 mV. (D) Summary of differential effects of rapalogs on *I*_TRPML1_. Data are presented as mean ± SEM. (E) Rap and rapalogs inhibited mTOR activity, which was assayed by phosphorylation of the mTOR substrate S6K at Thr 389. (F) Rap activated *I*_TRPML1_ in cells transfected with WT mTOR (left) and a kinase-dead mTOR^D2357E^ mutant (right). (G) mTOR mutants did not alter Rap sensitivity of *I*_TRPML1_. Data are presented as mean ± SEM. (H) Rap activated *I*_TRPML1_ in both *WT* (left) and *TSC2* KO (mTOR constitutively active, right) MEF cells. Inset shows the lack of TSC2 proteins in the *TSC2* KO. (I) Rap effects on *I*_TRPML1_ in *RagA* and *B* KO (mTOR deficient, right) MEF cells. Inset shows the lack of RagA proteins in the *RagA* and *B* KO. (J) Rap activated larger endogenous *I*_TRPML1_ in *p18* KO (right) compared with *WT* (left) HEK293 cells. Inset shows the lack of p18 proteins in the *p18* KO. Note that in *p18* KO cells, endogenous TFEB was localized in the nucleus, presumably due to mTOR deficiency (see S2K Fig), which in turn increased *I*_TRPML1_, because *TRPML1* is the one of major target genes of TFEB [10]. Only representative data are presented in A–C, F, and H–J. The individual data underlying D and G can be found in S1 Data. CTRL, control; Defo, deforolimus; Eve, everolimus; HEK293, human embryonic kidney 293 cell; KO, knockout; MEF, mouse embryonic fibroblast; mTOR, mechanistic target of rapamycin; p18, late endosomal/lysosomal adaptor, MAPK and mTOR activator 1 (LAMTOR1); Rag, Ras-related GTP-binding protein; Rap, rapamycin; Seco, seco-rapamycin; S6K, S6 kinase; Tem, temsirolimus; TFEB, transcription factor EB; Thr 389, threonine 389; TRPML1, transient receptor potential channel mucolipin 1; TSC2, tuberous sclerosis complex 2; WT, wild type; Zota, zotarolimus.

Lysosome-localized mTOR is a well-established target of Rap [13], and mTOR inhibition reportedly modulates the lysosomal TPC Na^+^ channel [28] and TRPML1 [29] activities. However, we found that Rap (or ML-SA1) activation of *I*_TRPML1_ occurred in the presence or absence of ATP magnesium salt (Mg-ATP) in the cytoplasmic (bath) solution (Figs 1C, S2A–S2C), arguing against the involvement of mTOR. As a positive control, whole-endolysosome *I*_TPC2_ was confirmed to be sensitive to Mg-ATP (S2F Fig). We further examined whether other mTOR inhibitors, including Torin-1, a potent catalytic mTOR inhibitor that is structurally different from Rap (S1 Fig) [30], could activate *I*_TRPML1_. No noticeable activation was seen with various concentrations of Torin-1 (10 μM; see Fig 2A and 2D), which abolished mTOR activity completely in biochemical assays with an S6K phosphorylation readout (Fig 2E). These differential effects of Rap and Torin-1 suggest that Rap-induced TRPML1 activation is distinct from its inhibitory effect on mTOR.

The TRPML1 activation effects of several commercially available mTOR-inhibiting rapalogs (S1 Fig) were found to differ drastically (Fig 2E). Whereas Tem and Eve activated *I*_TRPML1_ readily, albeit with slightly lower potencies than Rap (Figs 2B, 2D and S1A), activation was not seen with Defo or Zota (Figs 2C, 2D and S1B). Furthermore, Seco-Rap, an open-ring metabolite of Rap, failed to activate *I*_TRPML1_ (Figs 2C, 2D and S1C). This dissociation of TRPML1 activation from mTOR suggests that Rap and rapalogs activate TRPML1 independent of mTOR inhibition.

### mTOR kinase activity is not required for Rap activation of TRPML1

To further rule out mTOR involvement in Rap activation, we adopted a genetic approach to abolish mTOR catalytic activity through the overexpression of a kinase-dead dominant-negative mutation (D2357E) of mTOR [31]. Consistent with previous reports [28, 32], Mg-ATP–induced *I*_TPC2_ suppression was largely abrogated in COS1 cells overexpressing mTOR^D2357E^ compared with cells transfected with WT mTOR (S2G Fig). In contrast, mTOR^D2357E^ overexpression did not alter Rap-induced *I*_TRPML1_ (Fig 2F and 2G). The robust stimulatory effect of Rap on *I*_TRPML1_ was retained in cells overexpressing either a Rap-insensitive (S2035T) or a hyperactive (L1460P) mTOR mutant [33] (S2D and S2E Fig). Furthermore, Rap also robustly activated *I*_TRPML1_ in mTOR constitutively active (tuberous sclerosis complex 2 gene knockout [*TSC2* KO]) mouse embryonic fibroblasts (MEFs; Fig 2H and S2H Fig), as well as in mTOR-deficient Ras-related GTP-binding protein A and B gene double KO (*Rag A/B* KO) MEFs (Fig 2I and S2I Fig) and *p18/LAMTOR1* (late endosomal/lysosomal adaptor, MAPK and mTOR activator 1) gene KO human embryonic kidney 293 (HEK293) cells (Fig 2J and S2J Fig). Hence, Rap activates TRPML1 independent of mTOR activity.

We also generated mutations at mouse TRPML1 serine (Ser) 571 and Ser 576, residues corresponding to the mTOR-mediated phosphorylation sites (Ser 572 and Ser 576) in the human homolog [34]. Both nonphosphorylatable mutants (S571A/S576A) and phosphorylation-mimicking mutants (S571D/S576D) of TRPML1 were activated readily by Rap or ML-SA1 (S2L Fig), further supporting the notion that Rap activation of TRPML1 is independent of mTOR kinase activity.

### Rap binds directly to TRPML1

**Fig 3:**
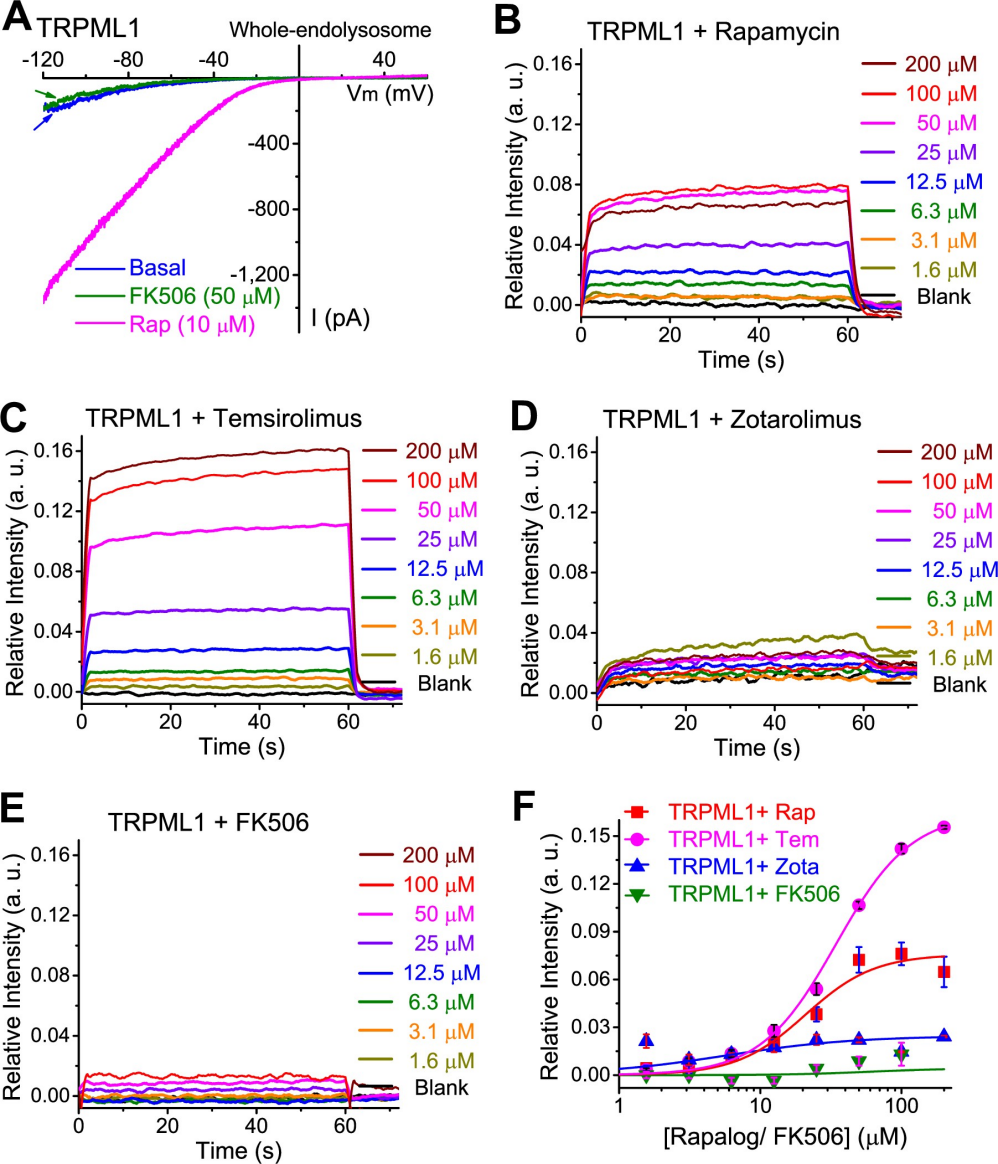
Rap and rapalogs bind TRPML1 in vitro. (A) Lack of FK506 effect on *I*_TRPML1_. Representative *I*_TRPML1_ was shown. (B) Rap bound to immuno-purified EGFP-TRPML1 immobilized on Pro-A biosensors in a dose-dependent manner. (C) Dose-dependent Tem-TRPML1 binding. (D) Weak or nonspecific binding of Zota to TRPML1. (E) Weak or nonspecific binding of FK506 to TRPML1. Panels B–E show representative binding activity from at least 4 independent experiments. (F) Dose-dependent Rap- and rapalog-TRPML1 binding. To avoid the interference of other Rap-targeting proteins, e.g., mTOR, we subtracted Rap binding activity in nontransfected HEK293 cells from that in EGFP-TRPML1–overexpressing cells. Data are presented as mean ± SEM (*n* = 4–6 independent experiments), and the individual data can be found in S1 Data. a.u., arbitrary unit; EGFP, enhanced green fluorescent protein; FK506, tacrolimus; HEK293, human embryonic kidney 293; mTOR, mechanistic target of rapamycin; Pro-A, protein A; Rap, rapamycin; Tem, temsirolimus; TRPML1, transient receptor potential channel mucolipin 1; Zota, zotarolimus.

We next performed biomolecular interaction analyses [3] to investigate the direct interaction between Rap and TRPML1. Unlike Rap, FK506 (Tacrolimus, a Rap analog) failed to activate TRPML1 channels (Fig 3A) and was thus used as a negative control. Immobilized FKBP12 on biosensor chips was used as a positive control [3]. Consistent with previous studies [3], sensorgrams displayed high-affinity binding (nM range K_D_) of Rap and FK506 with FKBP12 (S3C and S3D Fig). EGFP-TRPML1 proteins were immuno-purified with anti–green fluorescent protein (GFP) antibody (S3A Fig, inset) and immobilized on the protein A (Pro-A) sensor. Compared with the FK506 controls, TRPML1 proteins showed significant Rap binding with an estimated K_D_ = 20.9 ± 1.8 μM (*n* = 6 independent experiments; Fig 3B and 3E and 3F). Consistent with the electrophysiological analyses (Fig 2A–2C), Tem, but not Zota, also exhibited specific binding responses to TRPML1 (Fig 3C and 3D and 3F). Together, these in vitro interaction assay results suggest direct, specific bindings of Rap and rapalogs to TRPML1. The estimated in vitro binding affinity was roughly consistent with our electrophysiological results (see Fig 1C and 1D).

### Rap and/or Tem induces Ca^2+^-dependent TFEB nuclear translocation in TRPML1-overexpressing HeLa cells

**Fig 4:**
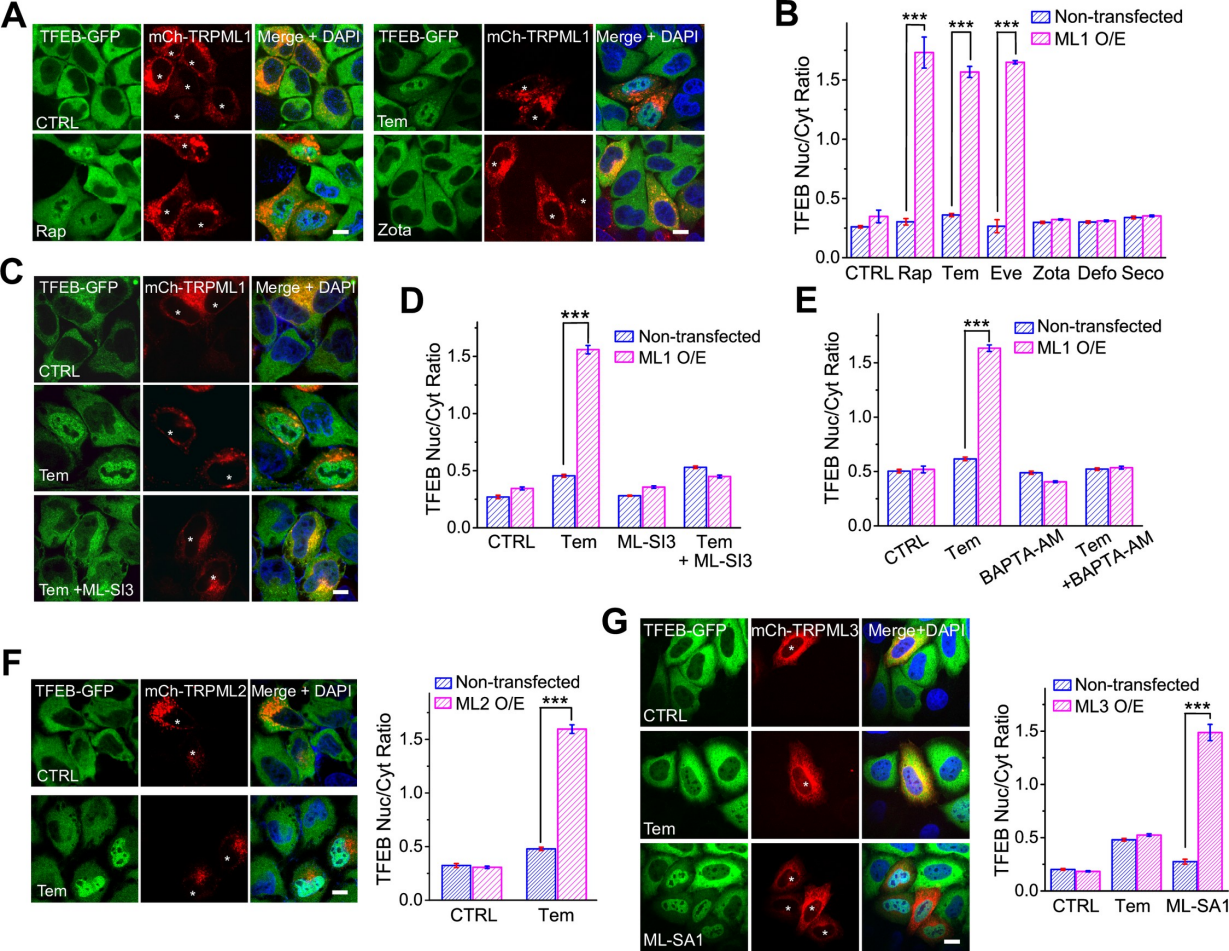
Rap and rapalogs induce TRPML1- and Ca^2+^-dependent TFEB nuclear translocation in TRPML1-overexpressing cells. (A) Rap (5 μM) and Tem (5 μM) induced TFEB nuclear translocation in TFEB-GFP stable cells overexpressing mCherry-TRPML1 (asterisks). TFEB nuclear translocation was not seen with Zota (5 μM). Scale bar = 10 μm. (B) Summary of rapalog effects on TFEB nuclear translocation. (C) Blockade of Tem-induced TFEB translocation by ML-SI3 (10 μM). Scale bar = 10 μm. (D) Quantification of ML-SI3 effect. (E) BAPTA-AM (5 μM, 1 h pretreatment) blocked Tem-induced TFEB nuclear translocation. (F) Tem (5 μM) induced TFEB nuclear translocation in TFEB-GFP stable cells overexpressing mCherry-TRPML2. Quantification is shown in the right panel. (G) The effects of Tem (5 μM, 2 h) and ML-SA1 (5 μM, 2 h) on TFEB nuclear translocation in TFEB-GFP stable cells that were transfected with mCherry-TRPML3. Data are quantified in the left panel. mCherry-positive cells are indicated by asterisks. Scale bar = 10 μm. Data shown in B and D–G were obtained from 30 to 40 cells from at least 3 independent experiments and are presented as mean ± SEM. The individual data supporting B and D–G can be found in S1 Data. ***P < 0.001, one-way ANOVA. BAPTA-AM, 1,2-Bis(2-aminophenoxy)ethane-N,N,N’,N’-tetraacetic acid tetrakis (acetoxymethyl ester); CTRL, control; Cyt, cytoplasm; Defo, deforolimus; Eve, everolimus; GFP, green fluorescent protein; mCh, mCherry; mCherry, monomeric red fluorescent protein; ML1, TRPML1; ML-SA1, TRPML1 synthetic agonist 1; ML-SI3, TRPML1 synthetic inhibitor 3; Nuc, nuclear; O/E, overexpression; Rap, rapamycin; Seco, seco-rapamycin; Tem, temsirolimus; TFEB, transcription factor EB; ML1/TRPML1, transient receptor potential channel mucolipin 1; Zota, zotarolimus.

Recently, we showed that TRPML1 activation by ML-SAs and reactive oxygen species is sufficient to activate TFEB (via nuclear translocation) and enhance autophagy in a Ca^2+^-dependent but mTOR-independent manner [22]. On Henrietta Lacks (HeLa) cells stably expressing TFEB-GFP (TFEB stable cells), we found that low micromolar concentrations of Rap failed to induce TFEB nuclear translocation (Fig 4A and 4B). In TFEB stable cells overexpressing monomeric red fluorescent protein (mCherry)-TRPML1, however, Rap (5 μM) induced rapid, dramatic TFEB nuclear translocation (Fig 4A and 4B). Consistent with our electrophysiology data, TRPML1-activating rapalogs, such as Tem (5 μM) and Eve (5 μM), caused TFEB nuclear translocation, whereas nonactivating rapalogs did not (Fig 4A and 4B and S4A Fig). Endogenous TFEB was also activated by Rap or Tem, but not Zota, in TRPML1-overexpressing HeLa cells (S4C Fig). Note that Tem, a synthetic Rap ester [35], was more effective than Rap in TFEB nuclear translocation (S5B–S5E Fig), suggesting that certain chemical properties of Tem might have made it more suitable for cell-based assays. Tem-induced TFEB activation was abolished by coapplication of ML-SI3 (Fig 4C and 4D). Consistently, Tem failed to induce TFEB nuclear translocation in cells transfected with TRPML1^DD/KK^ (a channel-dead pore mutant; S4D and S4E Fig), whereas overexpression of a constitutively active mutant of TRPML1 (TRPML1^Va^) led to nuclear accumulation of TFEB (S4D and S4E Fig) in the absence of Tem. Hence, Rap and Tem activated TFEB in cells with relatively high expression levels of TRPML1. Finally, in agreement with our electrophysiology analyses (Fig 1I and 1J), Tem evoked TFEB nuclear translocation in TRPML2-transfected cells but not in TRPML3-transfected cells (Fig 4F and 4G).

Because TRPML1 is the major lysosomal Ca^2+^-release channel, we investigated whether Rap- and/or Tem-induced TFEB activation was Ca^2+^ dependent. Application of 1,2-Bis(2-aminophenoxy)ethane-N,N,N’,N’-tetraacetic acid tetrakis (acetoxymethyl ester) (BAPTA-AM), a membrane-permeable form of Ca^2+^ chelator, blocked Tem-induced TFEB activation (Fig 4E and S4B Fig). Consistently, Tem readily increased cytosolic Ca^2+^ levels in HEK293 cells that were stably expressing genetically encoded GFP- and calmodulin-based Ca^2+^ probe 7 (GCaMP7)-TRPML1 (S4G Fig), and the increases were blocked by ML-SI3 (S4G Fig). Tem also significantly increased cytosolic Ca^2+^ levels in TRPML2-transfected HEK293 cells (S4H Fig). Hence, consistent with the electrophysiological analyses (Fig 1I and 1J) and TFEB nuclear translocation assays (Fig 4F and 4G), Rap and/or Tem activates TRPML1 and TRPML2 but not TRPML3. Collectively, these results suggest that Rap and/or Tem activates TFEB via a TRPML1/2- and Ca^2+^-dependent mechanism.

### Rap and Tem activate TFEB through TRPML1 in human fibroblasts

**Fig 5:**
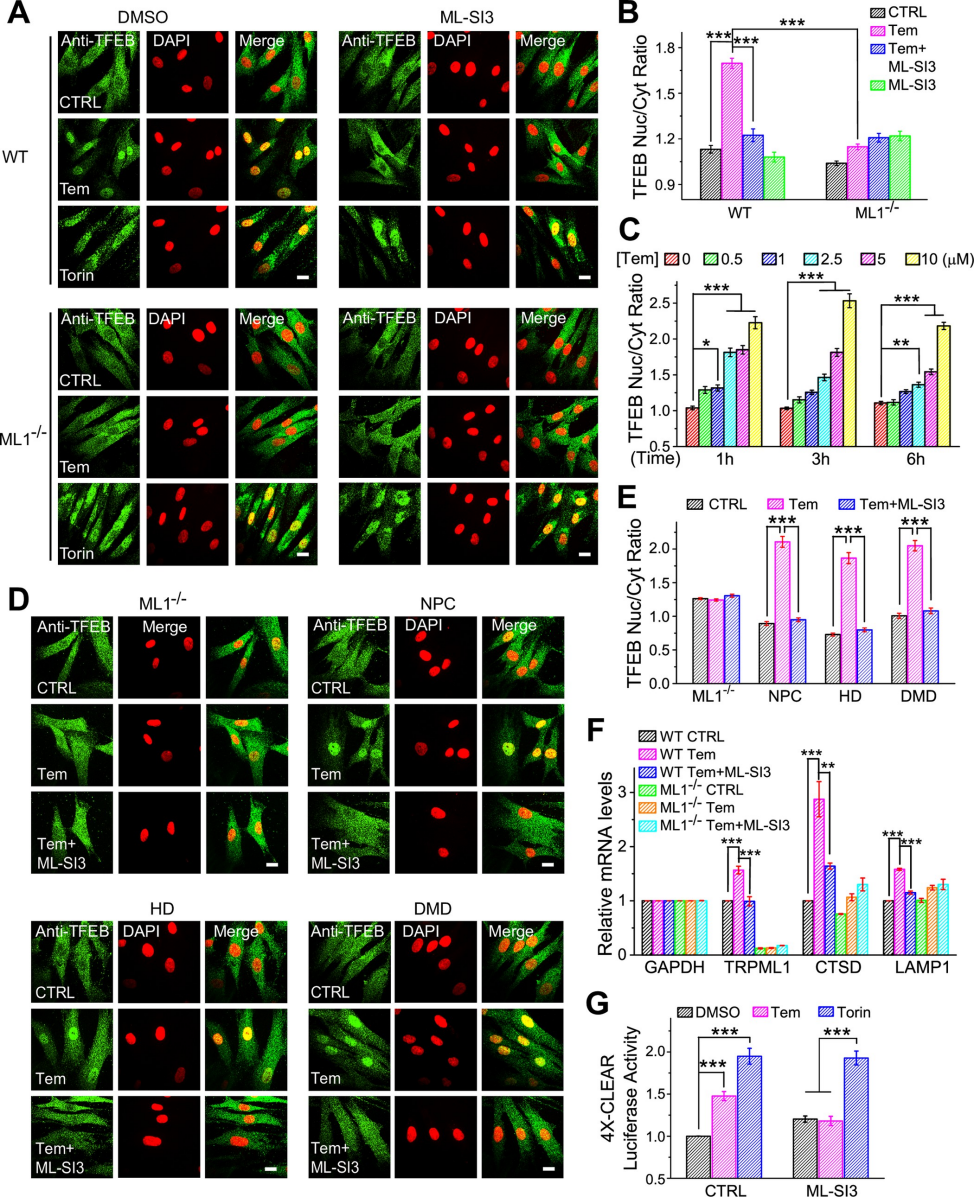
Tem activates the endogenous TRPML1-TFEB pathway. (A) Tem (10 μM, 9 h) induced TFEB (green) nuclear translocation in *WT* but not *ML1*^−/−^ fibroblasts. TFEB nuclear translocation was inhibited by coapplication of ML-SI3 (10 μM). Nuclei were labelled with DAPI (red, pseudo-color). Scale bar = 10 μm. (B) Summary of Tem effects on TFEB nuclear translocation in *WT* and *ML1*^−/−^ human fibroblasts. (C) Dose-dependent and time-dependent effects of Tem on TFEB translocation. (D) The effects of Tem (10 μM, 6 h) on cells derived from human disease tissues, e.g., *ML1*^−/−^, NPC, HD, and DMD. (E) Quantification of Tem effects shown in (D). Data shown in B, C, and E were obtained from more than 40 cells from at least 3 independent experiments. (F) The effects of Tem (10 μM, 16 h) on mRNA expression levels of *TRPML1*, *CTSD*, and *LAMP1* (*n* = 3–5 independent experiments). (G) The effects of Tem (10 μM, 16 h) on TFEB activity, measured using a 4X-CLEAR luciferase reporter (*n* = 4 independent experiments); Torin-1 (1 μM, 16 h) was used as a positive control. Data shown in B, C,and E–G are presented as mean ± SEM, and the individual data can be found in S1 Data. *P < 0.05, **P < 0.01, ***P < 0.001, one-way ANOVA. CTRL, control; CTSD, cathepsin D; Cyt, cytoplasm; DMD, Duchenne Muscular Dystrophy; GAPDH, Glyceraldehyde 3-phosphate dehydrogenase; HD, Huntington disease; LAMP1, lysosome-associated membrane protein 1; ML1^−/−^, Mucolipidosis IV; ML-SI3, TRPML1 synthetic inhibitor 3; NPC, Niemann-Pick type C; Nuc, nuclear; Tem, temsirolimus; TFEB, transcription factor EB; TRPML1, transient receptor potential channel mucolipin 1; WT, wild type; 4X-CLEAR, four CLEAR elements (GTCACGTGAC) in tandem derived from LAMP1 promoter + HTK.

Although several cell lines, such as HEK293 and HeLa cells, appeared to be “Rap-insensitive,” i.e., they lack Rap- and/or Tem-induced TFEB activation (S4I Fig), in multiple lines of *WT* human fibroblasts, 1 to 10 μM of Tem or 10 to 20 μM Rap robustly and quickly (within 1 h) activated TFEB (Fig 5A–5C and S5A–S5E Fig). The effects of Rap and Tem on TFEB nuclear translocation were abolished in Mucolipidosis IV (*ML1*^−/−^) human fibroblasts or by ML-SI3 (Fig 5A–5D and S5E Fig). In contrast, Torin-1–induced TFEB activation was unaffected (Fig 5A). Hence, Rap and Tem activated TFEB via TRPML1 in human fibroblasts. It is possible that the Rap-TRPML1-TFEB pathway was “sensitized” in human fibroblasts compared with other cell lines such as HEK cells. Notably, Tem (10 μM, 6 h) also induced dramatic TFEB nuclear translocation in multiple disease fibroblasts, including NPC fibroblasts, Huntington disease (HD) fibroblasts, and immortalized Duchenne Muscular Dystrophy (DMD) myoblasts (Fig 5D and 5E).

Calcineurin inhibitors, FK506 (5 μM) and cyclosporine (CsA, 10 μM) [21], reduced Tem-induced TFEB nuclear translocation (S5D and S5E Fig), suggesting that calcineurin may be the lysosomal Ca^2+^ sensor that mediates Rap activation of TFEB. TFEB nuclear translocation is determined by its phosphorylation status [18, 19]. TFEB phosphorylation at Ser 142 and Ser 211 was reduced by Rap and/or Tem in *WT* human fibroblasts, and the reduction was prevented by ML-SI3, *ML1*^−/−^ (S7A, S7B, S7D and S7E Fig), or by coapplication of FK506 and CsA (S7F and S7G Fig). Hence, the TRPML1-Ca^2+^-calcineurin pathway plays an essential role in Rap- and/or Tem-induced TFEB activation.

### Rap and Tem activate TFEB through TRPML1 to boost lysosomal functions

We next investigated the transcriptional activity of TFEB in TRPML1 stable HEK293 cells using a 4X-CLEAR luciferase reporter [36]. Tem (10 μM, 16 h) treatment increased 4X-CLEAR luciferase activity by approximately 50%, and the increase was suppressed by ML-SI3 (Fig 5G). Consistently, quantitative real-time polymerase chain reaction (RT-qPCR) analyses revealed that Tem (10 μM, 16 h) readily increased mRNA expression levels of TFEB target genes, including those related to lysosome biogenesis, e.g., *TRPML1*, cathepsin D (*CTSD*), and *LAMP1*, in a TRPML1-dependent manner (Fig 5F). Furthermore, both Rap (20 μM, 6 h) and Tem (10 μM, 6 h) treatment significantly increased the fluorescent intensities of both LysoTracker (an assay of lysosome acidification) and Magic Red (an assay of cathepsin B activity) in *WT* but not in *ML1*^-/-^ cells (S5F and S5G Fig). Taken together, these results suggest that Rap and Tem activation of TRPML1 may enhance lysosomal functions, e.g., by activating TFEB.

### Rap and Tem increase autophagic flux in a TRPML1-dependent manner

**Fig 6:**
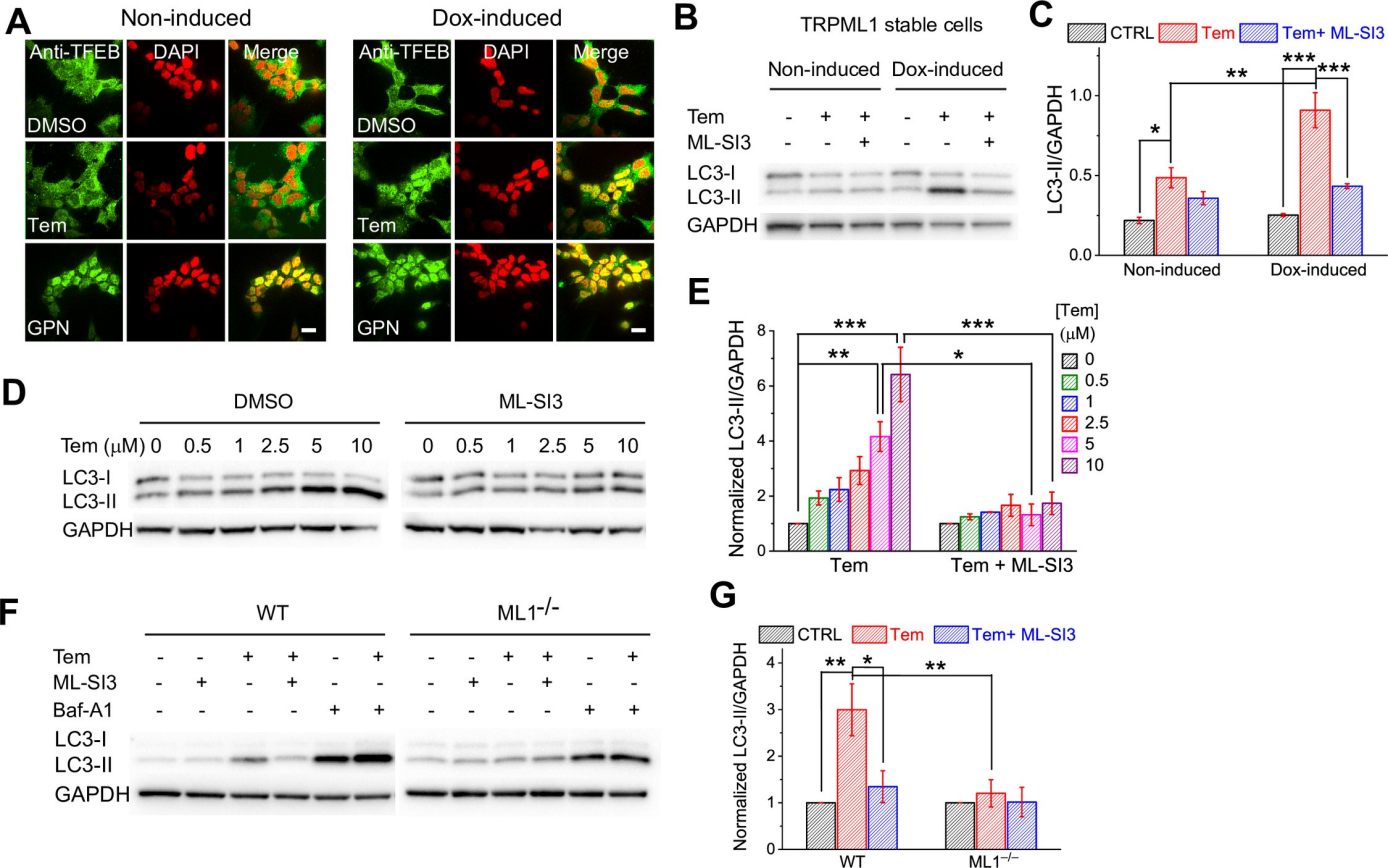
Tem increases autophagic flux through TRPML1. (A) Tem (5 μM, 3 h) induced TFEB (green) nuclear translocation in TRPML1 stable cell lines (TRPML1 HEK Tet-On) upon Dox (1 μg/ml, overnight) induction. GPN (200 μM, 2 h) was used as a positive control due to its consistent activation on TFEB in HEK cells [37]. Nuclei were labelled with DAPI (red, pseudo-color). Scale bar = 10 μm. (B) Tem (10 μM, 9 h) dramatically increased LC3-II levels in Dox-induced cells, which was blocked by ML-SI3 (10 μM). (C) Summary of Tem effects on LC3-II levels (normalized with GAPDH expression). (D) Dose-dependent effects of Tem (0.5, 1, 2.5, 5, and 10 μM; 9 h treatment) on LC3-II expression levels in Dox-induced TRPML1 stable cells. (E) Quantification of dose-dependent Tem effects shown in D. (F) Tem (10 μM, 9 h) elevated LC3-II levels in *WT* but not *ML1*^−/−^ human fibroblasts. Baf-A1 treatment increased LC3-II levels in both *WT* and *ML1*^−/−^ cells. Tem effects in *WT* cells were blocked by ML-SI3. (G) Quantification of Tem effects on LC3-II levels in fibroblasts. Data shown in C, E, and G were obtained from at least 3 independent experiments and are presented as mean ± SEM. The individual data of C, E, and G can be found in S1 Data. *P < 0.05, **P < 0.01, ***P < 0.001, one-way ANOVA. Baf-A1, Bafilomycin A1; CTRL, control; Dox, doxycycline; GAPDH, Glyceraldehyde 3-phosphate dehydrogenase; GPN, Glycyl-l-phenylalanine 2-naphthylamide; HEK, human embryonic kidney 293 cells; LC3-II, microtubule-associated proteins 1A/1B light chain 3B-II; ML^−/−^, Mucolipidosis IV; ML-SI3, TRPML1 synthetic inhibitor 3; Tem, temsirolimus; Tet-On, Tetracycline-On; TFEB, transcription factor EB; TRPML1, transient receptor potential channel mucolipin 1; WT, wild type.

In HEK293 cells, Tem (10 μM) induced clear TFEB nuclear translocation, but only when TRPML1 was overexpressed (Fig 6A). Hence, HEK293 cells are “Rap-insensitive” cells, in which the Rap-TRPML1-TFEB pathway can be sensitized with TRPML1 overexpression. Consistently, a dramatic increase in microtubule-associated proteins 1A/1B light chain 3B (LC3)-II protein levels was induced by Tem (10 μM, 9 h) in TRPML1 stable HEK293 cells upon doxycycline (Dox) induction; only mild effects were seen in noninduced cells (Fig 6B–6E).

In *WT* human fibroblasts in which the Rap-TRPML1-TFEB pathway is sensitized, Tem robustly increased LC3-II protein levels (Fig 6F and 6G, S6C and S6H and S6J Fig). Blocking lysosome function using the vacuolar H^+^-ATPase (V-ATPase) inhibitor, Bafilomycin A1 (Baf-A1), further increased LC3-II levels (Fig 6F and S6D Fig). In contrast, the Tem effects on LC3-II were abolished in *ML1*^*-/-*^ cells or by ML-SI3 or calcineurin inhibitors (Fig 6B–6G and S6C, S6H, S6J and S7F Figs). Tem also markedly increased LC3-II levels in cancer cell lines (S6E Fig). Likewise, potent ML-SA compounds had a similar effect (S6A Fig).

Likewise, Tem (10 μM, 2 h) significantly increased GFP-positive and red fluorescent protein (RFP)-positive (GFP^+^RFP^+^) puncta (autophagosome) in GFP-RFP-LC3 stable HeLa cells overexpressed with Cyan Fluorescent Protein (CFP)-TRPML1 (S6F and S6G Fig), which was largely diminished in the presence of ML-SI3 (S6F and S6G Fig). Sequestosome-1 (SQSTM1**/**p62) is another indicator of autophagic flux [38]. Whereas short-term (3–6 h) treatment of Tem slightly reduced p62 levels, longer (e.g., 9–16 h) treatment indeed increased p62 protein levels in WT but not in *ML1*^*-/-*^ or ML-SI3–pretreated *WT* cells (S6H–S6J Fig). The mRNA expression levels of p62 were significantly increased by Tem, and the increases were blocked by ML-SI3 (S6K Fig). Therefore, Tem may regulate both fast protein degradation and slow gene expression of p62. Collectively, these results suggest that Tem activation of TRPML1 facilitates both autophagic flux and autophagosome biogenesis.

Both targets of Rap, mTOR and TRPML1, are known to converge on TFEB phosphorylation and dephosphorylation [21, 22]. To segregate these two effects, we investigated the effect of Rap activation of TRPML1 on mTOR using other mTOR substrates, such as S6K and ULK1 [9], as the readout. For instance, mTOR-mediated phosphorylation at Ser 758 inactivates the ULK1 complex to impede autophagy initiation [39]. TRPML1 inhibitors did not affect the inhibitory effects of Tem on p-S6K and p-ULK1 levels (S6C and S7A–S7C Figs). In addition, Tem effects on LC3-II levels were also preserved in 5' adenosine monophosphate-activated protein kinase (AMPK) α1/α2 double KO MEFs (S6B Fig). Taken together, these results suggest that Rap and Tem increase autophagic flux mainly through TRPML1 activation instead of mTOR inhibition or AMPK activation, two well-known signaling pathways that mediate autophagy [12].

## Discussion

Rap and rapalog actions have been presumed to be mediated by inhibition of mTOR [4]. For instance, the neuroprotection and anti-aging effects of Rap have been attributed to its effects on autophagy induction [5]. Rap induction of autophagy has thus far been attributed to mTOR-mediated inhibition of ULK1 [4]. When mTOR is active, autophagy is inhibited by phosphorylation of the autophagy regulatory complex containing ULK1 [7]. However, autophagy induction alone is unlikely to increase autophagic flux given the severely compromised state of lysosome functions in many neurodegenerative diseases and aging [17]. Indeed, when lysosomes are dysfunctional, such as in various LSDs and neurodegenerative diseases, increased autophagic induction may further burden diseased cells, worsening pathological symptoms [17].

**Fig 7:**
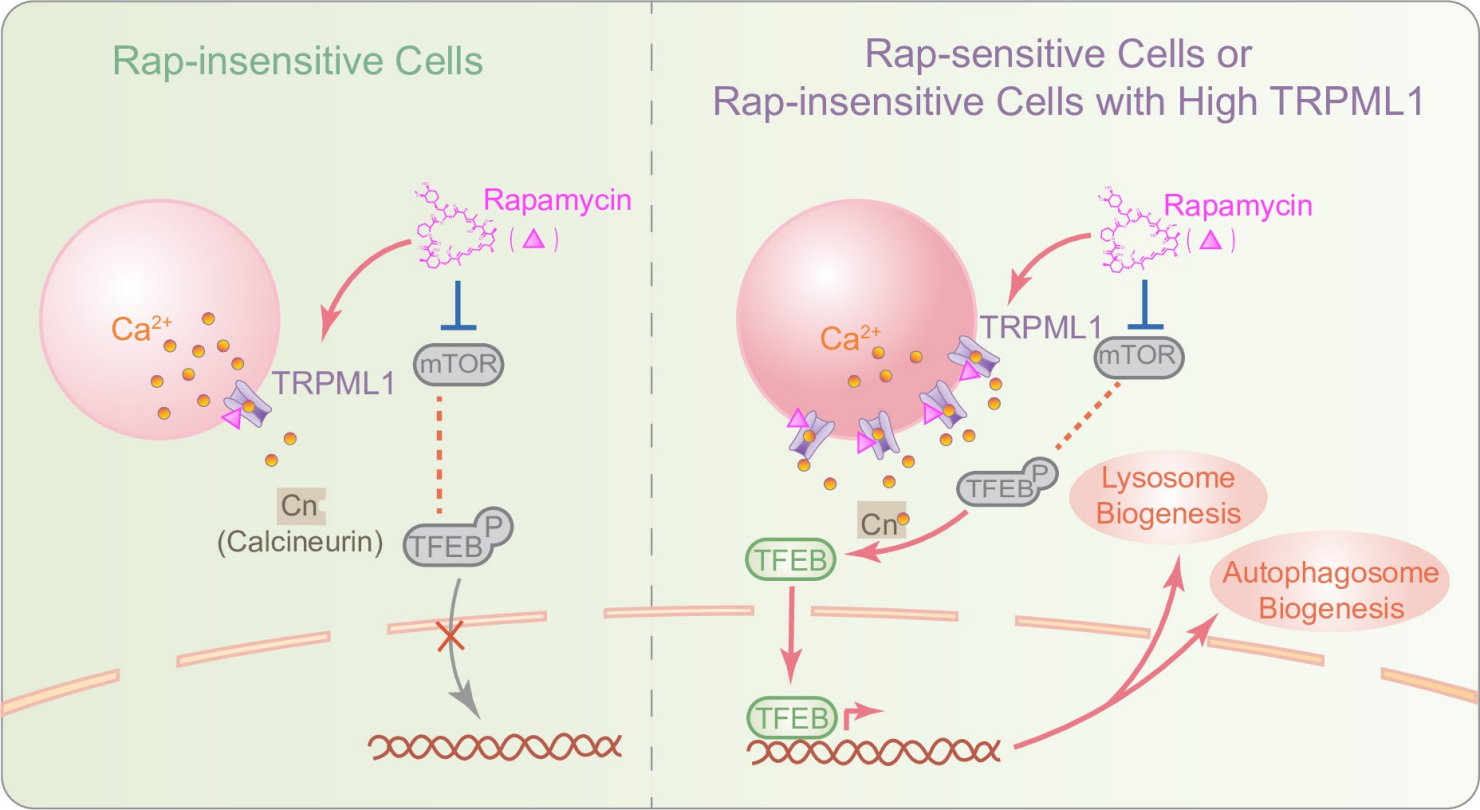
A working model of Rap stimulation of cellular clearance via the TRPML1-Ca^2+^-TFEB pathway. Rap effects are sensitive to TRPML1 expression levels in “Rap-insensitive” cells. When TRPML1 expression is low, mTOR is in an active state in which it phosphorylates and inactivates TFEB via cytosolic retention. Rap inhibition of mTOR is insufficient to cause TFEB nuclear translocation. In “Rap-sensitive” cells, in which the Rap-TRPML1-TFEB pathway is sensitized, or stressed cells with up-regulated TRPML1, Rap binds and activates TRPML1 channels, inducing substantial lysosomal Ca^2+^ release. Increases in perilysosomal Ca^2+^ levels activate Cn, causing TFEB translocation from the cytosol to the nucleus. Activated TFEB then promotes the expression of autophagic and lysosomal genes, enhancing the autophagic-lysosomal degradation pathway and cellular clearance. Cn, calcineurin; mTOR, mechanistic target of rapamycin; Rap, rapamycin; TFEB, transcription factor EB; TRPML1, transient receptor potential channel mucolipin 1.

The current study challenges the popular presumption that mTOR is the sole Rap target in the lysosome by demonstrating that the lysosomal Ca^2+^-permeable channel TRPML1 is also a target of Rap and/or rapalogs. Rap was shown to activate TRPML1 via direct binding, independent of its actions on mTOR. Unlike Rap-FKBP12 binding that displays a nanomolar affinity, the Rap-TRPML1 interaction has a much lower binding affinity. However, although nM concentrations of Rap and rapalogs robustly block the S6K phosphorylation, complete inhibition of 4E-BP requires much higher concentrations in normal cells (>500 nM) and certain cancer cells (>20 μM) [40]. Furthermore, the anti-neurodegeneration and anti-aging effects of Rap and/or rapalogs generally require higher doses of Rap, e.g., 5 to 20 μM via intraperitoneal injection [11]. Hence, in such *in vivo* studies, it is possible that the Rap-TRPML1 interaction in the micromolar range may induce lysosomal Ca^2+^ release and TFEB activation, especially in the cells with higher levels of TRPML1 expression and endogenous agonists (e.g., PI(3,5)P_2_ and reactive oxygen species [ROS]) [22]. TFEB nuclear translocation then induces the expression of a unique set of genes involved in autophagosome and lysosome biogenesis [15], enhancing autophagic cellular clearance [15, 17, 24, 25] (Fig 7). Our study reveals a TRPML1-dependent mechanism that links Rap to autophagy via a transcriptional mechanism (Fig 7). The TFEB-dependent mechanism may boost lysosome function in addition to autophagy induction. Hence, unlike the Rap-mTOR-ULK1 pathway, the Rap-TRPML1-TFEB pathway may boost both autophagosome and lysosome biogenesis, increasing autophagic flux and cellular clearance. The effect of Rap on TFEB and autophagy is most obvious in the “sensitized” cells, e.g., WT and disease human fibroblasts. In the “nonsensitized” cells, such as HEK293 and HeLa cells, TRPML1 overexpression readily imparts the “sensitivity” (Fig 7). Although the mechanisms underlying differential Rap sensitivity in various cells remain to be elucidated, the TRPML1-TFEB pathway may play a more dominant role in the neuroprotective and anti-aging effects of Rap than the mTOR-ULK1 pathway under stressed conditions, such as nutrient deprivation or LSD, in which TRPML1 expression is elevated [23, 25], and the levels of endogenous agonists, e.g., ROS, are increased [22].

Recent studies have suggested the existence of crosstalk mechanisms among autophagy processes, mTOR, TFEB, and lysosomal Ca^2+^ [21, 41]. As both mTOR and our newly identified Rap-TRPML1-Ca^2+^-calcineurin pathways converge on TFEB phosphorylation or dephosphorylation, it may prove difficult to separate these 2 effects, e.g., whether the Rap-mTOR pathway could be “sensitized” by the TRPML1-Ca^2+^-calcineurin pathway. However, it has been demonstrated that TRPML1 activation and lysosomal Ca^2+^ release indeed increased rather than decreased mTOR activity [21, 41–43]. In addition, Rap-mediated inhibition of mTOR, assayed by other substrates—e.g., S6K and ULK1—is not affected by ML1 KO or inhibition. Furthermore, previous studies have revealed that both overexpression of constitutively active TRPML1 and pharmacological activation of TRPML1 are sufficient to induce TFEB activation without causing any inhibition of mTOR [21, 22, 41]. Therefore, the simplest interpretation to the collective results is that Rap activates the TRPML1-TFEB pathway independent of mTOR.

Because mTOR KO may be lethal, to dissect out the contribution of TRPML1 to the in vivo actions of Rap, it might be necessary to perform neuroprotection or anti-aging studies in TRPML1 KO and overexpressing transgenic mice [22]. Meanwhile, it might prove helpful to compare the in vivo efficacies of TRPML1-activating versus -nonactivating rapalogs. The hydroxyl group(s) at C40, found in Rap, Tem, and Eve, are missing in TRPML1-nonactivating rapalogs (S1 Fig). Studying the rapalog-TRPML1 interaction may provide clues into how to develop new rapalogs to activate endolysosomal Ca^2+^-permeable TRPML channels specifically. Although Rap’s TRPML1 activation mechanism is unclear, the availability of TRPML1 and TRPML3 cryo-electron microscopy (cryo-EM) structures [44, 45] may help to identify Rap-TRPML1 interaction motif and/or site(s). The ML-SA1 binding pocket of TRPML1 is formed by the protein’s pore helix 1, transmembrane S5, and transmembrane S6 [44, 45]. It remains to be determined whether Rap also binds to this same region. Nevertheless, biochemically, the present identification of TRPML1 as an additional Rap target, independent of mTOR, may lead to a better mechanistic understanding of Rap effects on cellular clearance.

## Materials and methods

### Molecular biology

WT mTOR construct (plasmid #26603) was purchased from Addgene (Massachusetts, USA). Additional mTOR and TRPML1 mutants were generated with a quick-change lightning site-directed mutagenesis kit (Qiagen, Maryland, USA) according to the manufacturer’s instructions. All constructs were confirmed by DNA sequencing and western blotting.

### Mammalian cell culture

TFEB-GFP stable cell line was kindly provided by Shawn M. Ferguson [19]. *RagA/B* KO, *TSC2* KO, and their *WT* control MEF cells were generous gifts from Drs. Kunliang Guan [46] and David Kwiatkowski [47], respectively. *p18/LAMTOR1* CRISPR KO cells were generated in HEK293 cells using the CRISPR/Cas9 system. The 20-nucleotide guide sequence (5ʹ- CTGCTACAGCAGCGAGAACG) targeting human *p18* gene was designed using the CRISPR design tool (http://crispr.mit.edu/). The single guide RNAs (sgRNA) encoding target nucleotides were cloned into a bicistronic expression vector, LentiCRISPR version 2 (a gift from Dr. Feng Zhang; Addgene plasmid #52961, Massachusetts, USA) [48]. HEK293 cells were then transfected with sgRNA-LentiCRISPR version 2 using lipofectamine 2000 (Thermo Fisher Scientific, New York, USA) and selected with 3 μg/ml puromycin for 24 h. After single-cell clones were established, their genomic DNAs were sequenced to confirm the intended genetic disruptions. The following human fibroblasts were obtained from Coriell Institute (New Jersey, USA): WT (GM08399 and GM00969), ML1^-/-^ (GM02048), NPC (GM18453), and HD (GM04281).

Unless otherwise indicated, all cell cultures were maintained in Dulbecco's modified Eagle medium supplemented with 10% fetal bovine serum (sometimes tetracycline-free) at 37°C in a humidified 5% CO_2_ incubator. Cells usually were split 1 d before the experiments and reached 50% to 70% confluency at the experiment day. Cells were transfected with 1 to 4 μg plasmids using lipofectamine 2000 (Thermo Fisher Scientific, New York, USA). Culture media were refreshed 4 to 6 h post transfection, and cells were subject to imaging or electrophysiology 36 to 48 h after transfection. To induce TRPML1 or GCaMP7-TRPML1 expression in TRPML1 stable cell lines (TRPML1 HEK Tet-On), 1 μg/ml of Dox was added to the culture medium for overnight.

### Confocal imaging

For TFEB immunofluorescence detection, cells grown on glass coverslips were fixed with 4% paraformaldehyde and permeabilized with 0.3% Triton X-100. They were blocked with 1% bovine serum albumin in phosphate buffered saline (PBS). Endogenous TFEB was detected with anti-TFEB primary antibody (1:200; Cell Signaling Technology, Massachusetts, USA) and antirabbit secondary antibodies conjugated to Alexa Fluor 488 (Thermo Fisher Scientific, New York, USA). Coverslips were mounted on slides with Fluoromount-G (Southern Biotech, Alabama, USA), and images were acquired with an Olympus Spinning-Disk confocal microscope.

### RNA extraction and RT-qPCR

Total RNA was extracted and purified from the cultured human fibroblasts using E.Z.N.A. HP total RNA kit (Omega Bio-tek, Georgia, USA). The cDNA was then synthesized using a Superscript III RT kit (Thermo Fisher Scientific, New York, USA). PCR mixture was prepared with PowerUp SYBR green 2X master mix (Thermo Fisher Scientific, New York, USA) using the following primers [21]: GAPDH, forward (fw): 5ʹ-tgcaccaccaactgcttagc-3ʹ, reverse (rev): 5ʹ-ggcatggactgtggtcatgag-3ʹ; TRPML1, fw: 5ʹ-gagtgggtgcgacaagtttc-3ʹ, rev: 5ʹ-tgttctcttcccggaatgtc-3ʹ; CTSD, fw: 5ʹ-cttcgacaacctgatgcagc-3ʹ, rev: 5ʹ-tacttggagtctgtgccacc-3ʹ; LAMP1, fw: 5ʹ-acgttacagcgtccagctcat-3ʹ, rev: 5ʹ-tctttggagctcgcattgg-3ʹ; and p62/SQSTM1, fw: 5ʹ-gcactaccgcgatgaggac-3ʹ, rev: 5ʹ-gcacttgtagcgggttccta-3ʹ. Real time qPCR was performed with ABI StepOnePlus Real-Time PCR System.

### Western blotting

Cells were lysed with ice-cold RIPA buffer (Boston BioProducts, Massachusetts, USA) in the presence of 1× protease inhibitor cocktail (Sigma, Missouri, USA) and phosphatase inhibitor cocktail 2 (Sigma, Missouri, USA), NaF (1 mM), and Na_3_VO_4_ (1 mM). Protein samples (10–100 μg) were then loaded and separated on 4% to 12% gradient sodium dodecyl sulfate (SDS)-polyacrylamide electrophoresis gels (Thermo Fisher Scientific, New York, USA) and transferred to polyvinylidene difluoride membranes. The membranes were blocked with 1% bovine serum albumin or 5% milk in PBS supplemented with 0.1% Tween20 for 1 h and then incubated with primary antibodies against S6K (1:1,000; Cell Signaling Technology, Massachusetts, USA), p-S6K (1:1,000; Cell Signaling Technology, Massachusetts, USA), GAPDH (1:5,000; Millipore, Massachusetts, USA), LC3 (1:1,000; Sigma, Missouri, USA), TFEB (1:1,000, Millipore, Massachusetts, USA), pS211-TFEB (1:500; Cell Signaling Technology, Massachusetts, USA), pS142-TFEB (1:1,000; Cell Signaling Technology, Massachusetts, USA), ULK1 (1:1,000; Cell Signaling Technology, Massachusetts, USA), and pS757-ULK1 (equivalent to human S758, 1:1,000; Cell Signaling Technology, Massachusetts, USA). Bound antibodies were detected with horseradish peroxidase-conjugated antirabbit or antimouse secondary antibodies (1:5,000) and enhanced chemiluminescence reagents (Thermo Fisher Scientific, New York, USA). The total S6K, ULK1, and TFEB were reblotted in the same membranes after stripping using a stripping buffer (Thermo Fisher Scientific, New York, USA) for 10 to 30 min. Protein levels were quantified with ImageJ (NIH) software. The LC3-II/GAPDH, p-ULK1/ULK1, and p-TFEB/TFEB ratios were further normalized to DMSO control of *WT* cells.

### Ca^2+^ imaging

GCaMP imaging was performed in HEK293 cells stably expressing GCaMP7-TRPML1, a lysosome-targeted genetically encoded Ca^2+^ sensor [25] or HEK293 cells overexpressing GCaMP3-TRPML1^DD/KK^ and mCherry-TRPML2. Fluorescence intensity at 488 nm was recorded with an EasyRatioPro system (Photon Technology International, Inc. New Jersey, USA).

### Immunopurification of EGFP-TRPML1

Nontransfected and EGFP-TRPML1–expressing HEK293 cells were lysed in an immunoprecipitation buffer that contained 50 mM Tris-HCl, 150 mM NaCl, 1% NP-40, 2 mM CaCl_2_ (pH 7.5), and 1× protease inhibitor mix. Lysates were centrifuged at 14,000*g* for 10 min, and supernatants were incubated with an anti-GFP antibody (GenScript, Jiangsu, China; 1 μg per 1 × 10^7^ cells) at 4°C for 1 h. Pro-A/protein G plus-agarose (Santa Cruz, Shanghai, China) was then added (10 μl per μg of antibody), and the mix was incubated at 4°C overnight with gentle shaking. Agarose beads were washed with the immunoprecipitation buffer 4 times, then used in Rap binding assays.

### Biomolecular interaction assay

FKBP12, a high-affinity Rap-binding protein, was used as an internal control [3]. Hexahistidine (his6)-tagged FKBP12 was purified and biotinylated and then immobilized on the streptavidin (SA) biosensors for 10 min [3]. Similarly, recombination TRPML1 (approximately 100 μg/ml) and HEK293 cell lysates (approximately 100 μg/ml) were immunopurified and were immobilized onto Pro-A biosensors. The compound-protein binding was determined by sequentially immersing individual biosensors into Rap- and/or rapalog-PBST buffer (containing PBS, 0.05% Tween 20, and 0.02% BSA) for 100 s at each concentration (1.6, 3.1, 6.3, 12.5, 25, 50, 100, and 200 μM). The compound-protein interaction was recorded and analyzed by Octet Bio-Layer Interferometry Systems (ForteBio, Shanghai, China).

### Whole-endolysosome electrophysiology

Experiments were performed in mechanically isolated endolysosomes as described previously [22, 26, 27]. In brief, cells were treated with 1 μM vacuolin-1 overnight to increase the size of late endosomes and lysosomes selectively [49], and TRPML2 and TRPML3 were recorded from vacuoles enlarged with 300 nM vicenistatin overnight [50]. Unless otherwise indicated, vacuoles were bathed continuously in an internal (cytoplasmic) solution containing 140 mM K^+^-gluconate, 4 mM NaCl, 1 mM EGTA, 2 mM MgCl_2_, 0.39 mM CaCl_2_, and 20 mM HEPES (pH adjusted with KOH to 7.2; free [Ca ^2+^]_i_ approximately equal to 100 nM). The pipette (luminal) solution contained 145 mM NaCl, 5 mM KCl, 2 mM CaCl_2_, 1 mM MgCl_2_, 10 mM glucose, 10 mM HEPES, and 10 mM MES (pH adjusted to 4.6 or 7.4 with NaOH). The whole-endolysosome configuration was achieved as described previously [26]. After formation of a giga-seal between the patch pipette and an enlarged endolysosome, voltage steps of several hundred millivolts with a millisecond duration were applied to break into the vacuolar membrane [26]. All bath solutions were applied via a fast perfusion system that produced a complete solution exchange within a few seconds. Data were collected via an Axopatch 2A patch clamp amplifier, Digidata 1440, and processed with pClamp 10.0 software (Axon Instruments, Molecular Device, California, USA). Whole-endolysosome currents were digitized at 10 kHz and filtered at 2 kHz. All experiments were conducted at room temperature (21°C–23°C), and all recordings were analyzed in pCLAMP10 (Axon Instruments, Molecular Device, California, USA) and Origin 8.0 software.

### LysoTracker staining

Lysosomal acidity was detected using LysoTracker Red DND-99 (L7528; Thermo Fisher Scientific, New York, USA). Briefly, human fibroblasts were split and cultured in a 24-well dish 1 d before the experiment. To visualize the acidic organelles, LysoTracker Red (50 nM) was added into the cell culture medium and incubated at 37°C for 30 min. Cells were then washed twice with PBS and kept in PBS for imaging. Images were taken using an Olympus IX81 inverted fluorescence microscope, and the intensity of LysoTracker was analyzed using ImageJ software.

### 4X-CLEAR luciferase assay

TFEB activity was measured in TRPML1 stable HEK293 cells using a dual-luciferase reporter system (Promega E1910, Wisconsin, USA). Briefly, cells were cotransfected with a 4X-CLEAR luciferase reporter (a gift from Dr. Albert La Spada; Addgene plasmid # 66800) [36] and Renilla luciferase plasmid in a 1:20 ratio for 6 h. Cells were lysed 24 h post transfection, and cell lysates were then transferred to a 96-well opaque plate. Luciferase activities were detected using GloMax Microplate Luminometer (Progema, Wisconsin, USA). The activity of 4X-CLEAR luciferase was divided by that of Renilla luciferase and then normalized to the DMSO controls.

### Cathepsin B activity assay

Cathepsin B activity was measured using Magic Red Cathepsin B assay kit (ImmunoChemistry Technologies, Minnesota, USA). Magic Red stock solution was prepared according to the manufacturer’s instruction. Cells were incubated with Magic Red reagent (1:1,000 dilution from stock solution) at 37°C for 1 h and fixed by 4% PFA before imaging. Images were taken using an Olympus IX81 inverted fluorescence microscope. Magic Red intensity was analyzed with ImageJ software.

### Reagents

Rap, Tem, and Eve were purchased from LC Laboratories (Massachusetts, USA) or MedChemExpress (New Jersey, USA). Defo (MK-86669) and Zota (ABT-578) were purchased from Selleckchem (Texas, USA). ML-SA1 was obtained from Princeton BioMolecular Research (New Jersey, USA). ML-SI3 was custom synthesized (available upon MTA request). Seco-Rap (148554-65-8) was from Cayman Chemical (Michigan, USA), Torin-1 was from Tocris (Minnesota, USA), BAPTA-AM was from Thermo Fisher (New York, USA), and vacuolin-1 was from Calbiochem (Millipore, Massachusetts, USA).

### Data analysis

Data are presented as means ± SEMs. Statistical comparisons of imaging results were performed with ANOVAs. *P* < 0.05 was considered statistically significant.

## Supporting information

S1 Fig Chemical structures of Rap and/or rapalogs. (A) Chemical structures of Rap and TRPML1-activating rapalogs. (B) Chemical structures of non-TRPML1–activating rapalogs. (C, D) Structure of Seco-Rap (C) and Torin-1 (D). Note that rapalogs differ at the C40 site (highlighted in red). (E) Synergistic effect of PI(3,5)P_2_ and Rap on TRPML1 activation. Rap-activated *I*_TRPML1_ was further enhanced in the presence of 0.1 μM of PI(3,5)P_2_. C40, carbon 40; PI(3,5)P_2_, phosphatidylinositol 3,5-bisphosphate; Rap, rapamycin; Seco, seco-rapamycin; TRPML1, transient receptor potential channel mucolipin 1.
(PDF)

S2 Fig Rap activation of *I*_TRPML1_ is independent of mTOR. (A) Rap activated *I*_TRPML1_ in the presence of Mg-ATP. (B) Addition of Mg-ATP (1 mM) to the bath solution did not inhibit Rap-evoked *I*_TRPML1_. (C) Mg-ATP also did not affect ML-SA1–induced *I*_TRPML1_. (D) Rap-activated whole-endolysosomal *I*_TRPML1_ in COS1 cells transfected with mTOR^S2035T^. (E) Rap-activated *I*_TRPML1_ in COS1 cells transfected with mTOR^L1460P^, a hyperactive mTOR mutant. (F) PI(3,5)P_2_-induced *I*_TPC2_ was suppressed by Mg-ATP (1 mM). (G) ATP effects on *I*_TPC2_ in cells overexpressing WT mTOR (left) or mTOR^D2357E^ mutant (middle), and the quantification of ATP effects (right). (H, I) Quantification of Rap effects on *I*TRPML1. (J) Rap effects on endogenous *I*_TRPML1_ in *p18* WT and KO cells. (K) CRISPR-Cas9 KO of *p18* caused constitutive activation (i.e., nuclear translocation) of TFEB (lower). (L) Stimulatory effect of Rap was retained in nonphosphorylatable TRPML1^S571A/S576A^ (left) and phosphorylation-mimicking TRPML1^S571D/S576D^ (right) mutant channels. Data shown in (G–J) are presented as mean ± SEM, and the individual data can be found in S1 Data. COS1, CV-1 in Origin Simian-1; CRISPR, Clustered Regularly Interspaced Short Palindromic Repeats; Cas9, caspase 9; KO, knockout; Mg-ATP, adenosine 5'-triphosphate magnesium salt; ML-SA1, TRPML1 synthetic agonist 1; mTOR, mechanistic target of rapamycin; p18, late endosomal/lysosomal adaptor, MAPK and MTOR activator 1; Rap, rapamycin; TFEB, transcription factor EB; WT, wild type.
(PDF)

S3 Fig In vitro Rap-TRPML1, Rap-FKB12 and FK506-FKB12 binding assays. (A) Weak binding of Rap with HEK293 lysates. Inset shows EGFP-TRPML1 (approximately100 kDa) immuno-purified with an anti-GFP antibody. Averaged binding activities from 6 independent experiments are shown. (B) Rap bound to immuno-purified EGFP-TRPML1 immobilized on Pro-A biosensors in a dose-dependent manner. Averaged binding activities from 6 independent experiments are shown. (C, D) Rap (C) and FK506 (D) bound to biotinylated FKBP12 (immobilized on the SA biosensors). Representative binding activity are shown. EGFP, enhanced green fluorescent protein; FK506, tacrolimus; FKB12, Peptidylprolyl isomerase; GFP, green fluorescent protein; HEK293, human embryonic kidney 293 cells; Pro-A, protein A; Rap, rapamycin; SA, streptavidin; TRPML1, transient receptor potential channel mucolipin 1.
(PDF)

S4 Fig Tem-induced TFEB nuclear translocation is Ca^2+^ and TRPML dependent. (A) Eve (5 μM, 2 h) induced TFEB nuclear translocation in TFEB-GFP stable cells overexpressing mCherry-TRPML1 (indicated by asterisks). In contrast, no obvious TFEB nuclear translocation was seen with Defo (5 μM, 2 h), Seco-Rap (5 μM), or ML-SI3 (10 μM). Scale bar = 10 μm. (B) BAPTA-AM (5 μM, 1 h pretreatment) blocked Tem-induced TFEB nuclear translocation. Scale bar = 10 μm. (C) Rap (5 μM, 2 h) and Tem (5 μM), but not Zota (5 μM), induced endogenous TFEB nuclear translocation in HeLa cells overexpressing mCherry-TRPML1 (indicated by asterisks). Scale bar = 10 μm. (D) Tem showed no effect on TFEB nuclear translocation in cells transfected with TRPML1^DD/KK^_,_ a channel-dead pore mutant (upper). Overexpression of constitutively active TRPML1^Va^ mutant resulted in nuclear accumulation of TFEB in the absence of Tem (lower). (E) Quantitation of TFEB nuclear translocation of (D) from 30 to 40 cells in 3 independent experiments. (F) The effects of ML-SI3 (10 μM, 1 h) pretreatment on ML-SA1– and Torin-1–induced TFEB nuclear translocation in TFEB-GFP stable cells that were transfected with mCherry-TRPML3 (indicated by asterisks). (G) Tem increased cytosolic Ca^2+^ levels through TRPML1 activation. In cells stably expressing GCaMP7-TRPML1, Tem (50 μM) and ML-SA1 (5 μM) increased GCaMP7 fluorescence intensity, which was blocked by ML-SI3 (10 μM) coapplication (left). Iono (1 μM) was used as a positive control. The effects of Tem were quantified from 9 independent experiments (right) and presented as mean ± SEM. (H) The effects of Tem (50 μM) on cytosolic Ca^2+^ levels in HEK293 cells that were cotransfected with mCherry-TRPML2 and GCaMP3-TRPML1^DD/KK^. (I) Tem (10 μM, 9 h) failed to induce TFEB (green) nuclear translocation in HEK293 and HeLa cells. Note that Torin-1 (1 μM) induced dramatic TFEB nuclear translocation in HeLa cells but mild TFEB nuclear translocation in HEK293 cells. Nuclei were labelled with DAPI (red, pseudo-color). Scale bar = 10 μm. The individual data underlying (E) and (G) can be found in S1 Data. BAPTA-AM, 1,2-Bis(2-aminophenoxy)ethane-N,N,N’,N’-tetraacetic acid tetrakis (acetoxymethyl ester); Defo, deforolimus; Eve, everolimus; GCaMP7, GFP- and calmodulin-based Ca^2+^ probe 7; GFP, green fluorescent protein; HEK293, human embryonic kidney 293 cells; HeLa, Henrietta Lacks cells; Iono, Ionomycin; mCherry, a monomeric red fluorescent protein; ML-SA1, TRPML1 synthetic agonist 1; ML-SI3, TRPML1 synthetic inhibitor 3; Rap, rapamycin; Seco, seco-rapamycin; Tem, temsirolimus; TFEB, transcription factor EB; TRPML1, transient receptor potential channel mucolipin 1; Zota, zotarolimus.
(PDF)

S5 Fig Rap- and Tem-induced TFEB nuclear translocation is TRPML1 dependent. (A) Dose- and time-dependent effects of Tem on TFEB nuclear translocation. Scale bar = 10 μm. (B) Rap and Tem effects on TFEB nuclear translocation in human fibroblasts. Scale bar = 10 μm. (C) Quantification of Rap and Tem effects shown in (B). (D, E) The effects of calcineurin inhibitors FK506 (5 μM) and CsA (10 μM) on Rap- and Tem-induced TFEB nuclear translocation in *WT* and *ML1*^*-/-*^ human fibroblasts. Scale bar = 10 μm. (F) Effects of Rap (20 μM, 6 h) and Tem (10 μM, 6 h) on LysoTracker staining in *WT* and *ML1*^*-/-*^ human fibroblasts. Torin-1 (1 μM, 6 h) was used as a control. Scale bar = 100 μm. (G) The effects of Rap (20 μM, 6 h) and Tem (10 μM, 6 h) on Magic Red staining in *WT* and *ML1*^*-/-*^ cells. Torin-1 (1 μM, 6 h) was used as a control. Scale bar = 100 μm. Averaged data shown in the left panels of (F) and (G) were from 3 independent experiments and are presented as mean ± SEM. ***P < 0.001, one-way ANOVA. The individual data underlying C, D, F, and G can be found in S1 Data. CsA, cyclosporine A; FK506, tacrolimus; ML1^−/−^, Mucolipidosis IV; Rap, rapamycin; Tem, temsirolimus; TFEB, transcription factor EB; TRPML1, transient receptor potential channel mucolipin 1; WT, wild type.
(PDF)

S6 Fig Tem increases autophagic flux through a TRPML1-dependent mechanism. (A) TRPML1 synthetic agonists ML-SA1 (10 μM, 4 h) and ML-SA5 (1 μM, 4 h) increased LC3-II levels in *WT* human fibroblasts, and the increase was suppressed by ML-SI3 (10 μM). (B) Tem effect in *AMPK α1/α2* double KO MEFs. (C) The effects of ML-SI3 (10 μM) on Tem-induced increases in the LC3-II levels and mTOR inhibition in *WT* human fibroblasts. (D) The effects of Baf-A1 (0.5 μM, 9 h) on Tem-induced LC3-II increases in *WT* human fibroblasts (left). (E) The effects of TRPML1 inhibitors on the Tem (10 μM, 9 h) in M12 (prostate cancer), CN34 (breast carcinoma), and MeWo (melanoma) cells. (F) Tem (10 μM, 2 h) significantly increased GFP^+^RFP^+^ puncta in GFP-RFP-LC3 stable HeLa cells overexpressing CFP-TRPML1. Tem effect was inhibited by ML-SI3 (10 μM). (G) Quantification of F from more than 20 CFP-positive cells for each treatment. (H) Tem (10 μM, 9 h) increased p62 levels in *WT* but not *ML1*^*-/-*^ human fibroblasts. Tem effects in *WT* cells were blocked by ML-SI3 (10 μM). (I) Quantification of H. (J) Time-dependent effects of Tem (10 μM) on p62 and LC3 protein levels. (K) The effects of Tem (10 μM, 16 h) and ML-SI3 (10 μM) on p62/SQSTM1 transcript levels, analyzed by RT-qPCR. The black framed boxes indicate images coming from separated gel runs. Data shown in G, I, and K were obtained from at least 3 independent experiments and are presented as mean ± SEM. The individual data can be found in S1 Data. *P < 0.05, **P < 0.01, ***P < 0.001, one-way ANOVA. AMPK, 5' adenosine monophosphate-activated protein kinase; Baf-A1, Bafilomycin A1; CFP, Cyan Fluorescent Protein; GFP, green fluorescent protein; HeLa, Henrietta Lacks cells; KO, knockout; LC3-II, microtubule-associated proteins 1A/1B light chain 3B-II; MEF, mouse embryonic fibroblast; ML1^−/−^, Mucolipidosis IV; ML-SA, TRPML1 synthetic agonist; ML-SI3, TRPML1 synthetic inhibitor 3; mTOR, mechanistic target of rapamycin; p62/SQSTM1, Sequestosome-1; RFP, red fluorescent protein; RT-qPCR, quantitative real-time polymerase chain reaction; Tem, temsirolimus; TRPML1, transient receptor potential channel mucolipin 1; WT, wild type.
(PDF)

S7 Fig Rap and Tem increase autophagic flux through TRPML1-TFEB-dependent mechanisms. (A) The effects of Rap, Tem, and ML-SI3 on p-ULK1 and pS142-TFEB. (B) The effects of Tem and ML-SI3 on pS211-TFEB. (C) Quantification of p-ULK1. (D) Quantification of Tem effect on pS211-TFEB with or without ML-SI3. (E) The effect of Tem, Rap, and Torin-1 (shown in boxes) on pS142-TFEB in *WT* and *ML1*^*-/-*^ human fibroblasts. (F) The effects of calcineurin inhibitors and Tem on pS142-TFEB. (G) Quantification of pS142-TFEB under various treatment conditions. Data shown in C, D, and G were obtained from at least 3 independent experiments and are presented as mean ± SEM. The individual data can be found in S1 Data. *P < 0.05, **P < 0.01, ***P < 0.001, one-way ANOVA. ML1^−/−^, Mucolipidosis IV; ML-SI3, TRPML1 synthetic inhibitor 3; N.S., not significant; pS142-TFEB, phospho-TFEB at Ser 142; pS211-TFEB, phospho-TFEB at Ser 211; p-ULK1, phospho-ULK1 at Ser 758; Rap, rapamycin; Tem, temsirolimus; TFEB, transcription factor EB; TRPML1, transient receptor potential channel mucolipin 1; WT, wild type.
(PDF)

S1 Data Individual numerical values underlying all summary data presented in the manuscript. (XLSX)

## Abbreviations

AMPK: 5' adenosine monophosphate-activated protein kinase
Baf-A1: Bafilomycin A1
BAPTA-AM: 1,2-Bis(2-aminophenoxy)ethane-N,N,N’,N’-tetraacetic acid tetrakis (acetoxymethyl ester)
CFP: Cyan Fluorescent Protein
COS1: CV-1 in Origin Simian-1
cryo-EM: cryo-electron microscopy
CsA: cyclosporine A
CTSD: cathepsin D
Defo: deforolimus
DMD: Duchenne Muscular Dystrophy
Dox: doxycycline
EGFP: enhanced green fluorescent protein
Eve: everolimus
FK506: tacrolimus
FKBP: FK506 binding protein
FRB: FKBP-rapamycin binding
fw: forward
GCaMP7: GFP- and calmodulin-based Ca^2+^ probe 7
GFP: green fluorescent protein
GPN: Glycyl-l-phenylalanine 2-naphthylamide
HD: Huntington disease
HEK293: human embryonic kidney 293 cells
HeLa: Henrietta Lacks cells
his6: Hexahistidine
ILS-920: a rapamycin derivative
KO: knockout
p18/LAMTOR1: late endosomal/lysosomal adaptor, MAPK, and mTOR activator 1
LC3-II: microtubule-associated proteins 1A/1B light chain 3B-II
LSD: lysosome storage disease
mCherry: a monomeric red fluorescent protein
MCOLN1: mucolipin 1
MEF: mouse embryonic fibroblast
Mg-ATP: adenosine 5'-triphosphate magnesium salt
ML1^-/-^: Mucolipidosis IV
ML-SA1: TRPML1 synthetic agonist 1
ML-SI3: TRPML1 synthetic inhibitor 3
mTOR: mechanistic target of rapamycin
mTORC: mammalian target of rapamycin complex
NPC: Niemann-Pick type C
PBS: phosphate buffered saline
PI(3,5)P_2_: phosphatidylinositol 3,5-bisphosphate
Pro-A: protein-A
rev: reverse
Rap: Rapamycin
Rag: Ras-related GTP-binding protein
RFP: red fluorescent protein
ROS: reactive oxygen species
RT-qPCR: quantitative real-time polymerase chain reaction
SA: streptavidin
SDS: sodium dodecyl sulfate
sgRNA: single guide RNA
SQSTM1**/**p62: Sequestosome-1
S6K: S6 kinase
Tem: temsirolimus
TFEB: transcription factor EB
TOR: target of rapamycin
TPC2: two-pore channel 2
TRPML1: transient receptor potential channel mucolipin 1
TSC2: tuberous sclerosis complex 2
ULK1: UNC-5–like autophagy activating kinase
V-ATPase: vacuolar H^+^-ATPase
WT: wild type
WYE-592: a rapamycin derivative
Zota: zotarolimus
4E-BP1: 4E binding protein 1
