# Supplementary material for: Rapamycin directly activates lysosomal mucolipin TRP channels independent of mTOR
Source: PLoS Biol. 2019 May 21;17(5):e3000252. doi: 10.1371/journal.pbio.3000252 (PMC6528971; doi:10.1371/journal.pbio.3000252)

A

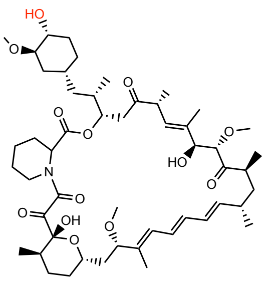

Rapamycin (Sirolimus)

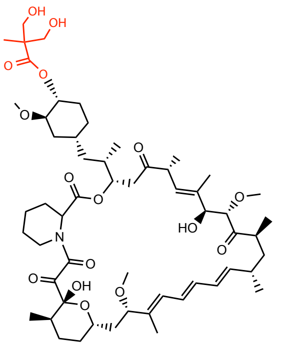

Temsirolimus

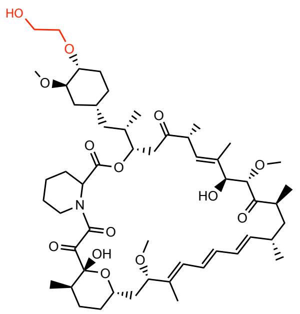

Everolimus

B

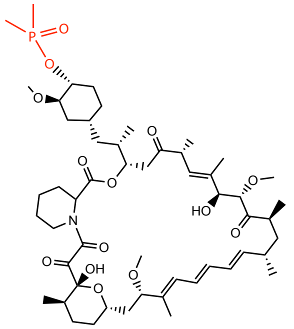

Deforolimus

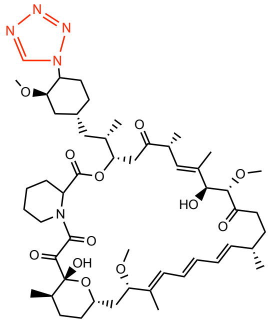

Zotarolimus

C

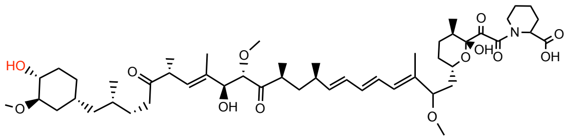

Seco rapamycin

D

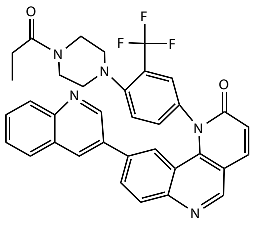

Torin-1

E

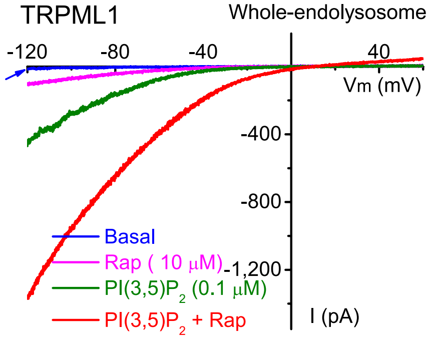

Supplement: S1 Fig — (A) Chemical structures of Rap and TRPML1-activating rapalogs. (B) Chemical structures of non-TRPML1–activating rapalogs. (C, D) Structure of Seco-Rap (C) and Torin-1 (D). Note that rapalogs differ at the C40 site (highlighted in red). (E) Synergistic effect of PI(3,5)P2 and Rap on TRPML1 activation. Rap-activated ITRPML1 was further enhanced in the presence of 0.1 μM of PI(3,5)P2. C40, carbon 40; PI(3,5)P2, phosphatidylinositol 3,5-bisphosphate; Rap, rapamycin; Seco, seco-rapamycin; TRPML1, transient receptor potential channel mucolipin 1. (PDF) [file pbio.3000252.s001.pdf]
