# Supplementary figures and images for: Rapamycin directly activates lysosomal mucolipin TRP channels independent of mTOR
Source: PLoS Biol. 2019 May 21;17(5):e3000252. doi: 10.1371/journal.pbio.3000252 (PMC6528971; doi:10.1371/journal.pbio.3000252)

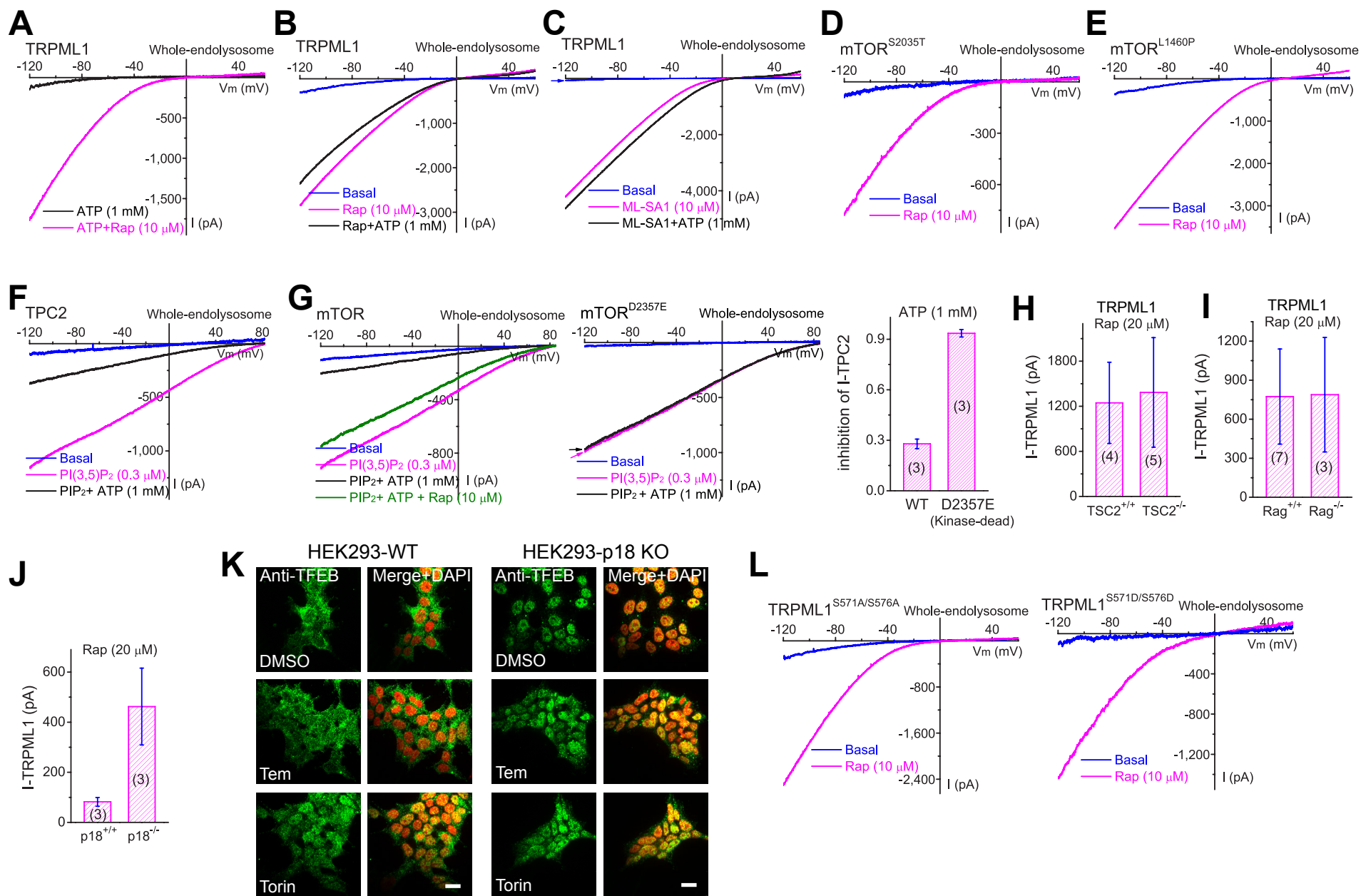

Supplement: S2 Fig — (A) Rap activated ITRPML1 in the presence of Mg-ATP. (B) Addition of Mg-ATP (1 mM) to the bath solution did not inhibit Rap-evoked ITRPML1. (C) Mg-ATP also did not affect ML-SA1–induced ITRPML1. (D) Rap-activated whole-endolysosomal ITRPML1 in COS1 cells transfected with mTORS2035T. (E) Rap-activated ITRPML1 in COS1 cells transfected with mTORL1460P, a hyperactive mTOR mutant. (F) PI(3,5)P2-induced ITPC2 was suppressed by Mg-ATP (1 mM). (G) ATP effects on ITPC2 in cells overexpressing WT mTOR (left) or mTORD2357E mutant (middle), and the quantification of ATP effects (right). (H, I) Quantification of Rap effects on ITRPML1. (J) Rap effects on endogenous ITRPML1 in p18 WT and KO cells. (K) CRISPR-Cas9 KO of p18 caused constitutive activation (i.e., nuclear translocation) of TFEB (lower). (L) Stimulatory effect of Rap was retained in nonphosphorylatable TRPML1S571A/S576A (left) and phosphorylation-mimicking TRPML1S571D/S576D (right) mutant channels. Data shown in (G–J) are presented as mean ± SEM, and the individual data can be found in S1 Data. COS1, CV-1 in Origin Simian-1; CRISPR, Clustered Regularly Interspaced Short Palindromic Repeats; Cas9, caspase 9; KO, knockout; Mg-ATP, adenosine 5'-triphosphate magnesium salt; ML-SA1, TRPML1 synthetic agonist 1; mTOR, mechanistic target of rapamycin; p18, late endosomal/lysosomal adaptor, MAPK and MTOR activator 1; Rap, rapamycin; TFEB, transcription factor EB; WT, wild type. (PDF) [file pbio.3000252.s002.pdf]

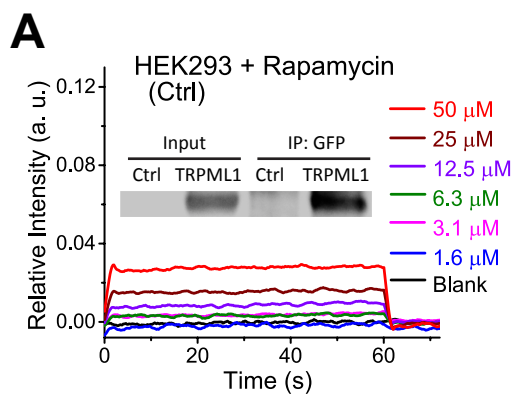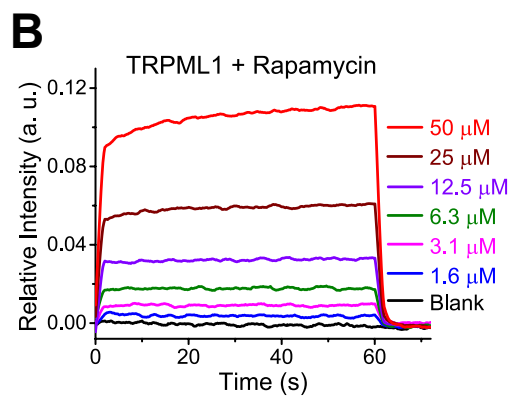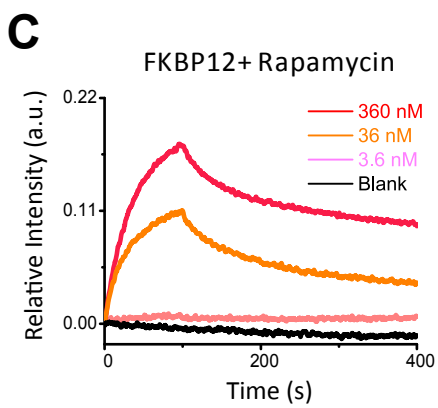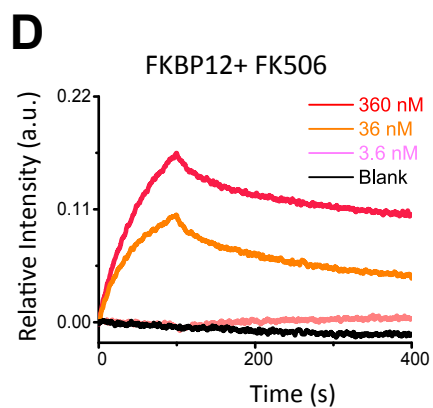

Supplement: S3 Fig — (A) Weak binding of Rap with HEK293 lysates. Inset shows EGFP-TRPML1 (approximately100 kDa) immuno-purified with an anti-GFP antibody. Averaged binding activities from 6 independent experiments are shown. (B) Rap bound to immuno-purified EGFP-TRPML1 immobilized on Pro-A biosensors in a dose-dependent manner. Averaged binding activities from 6 independent experiments are shown. (C, D) Rap (C) and FK506 (D) bound to biotinylated FKBP12 (immobilized on the SA biosensors). Representative binding activity are shown. EGFP, enhanced green fluorescent protein; FK506, tacrolimus; FKB12, Peptidylprolyl isomerase; GFP, green fluorescent protein; HEK293, human embryonic kidney 293 cells; Pro-A, protein A; Rap, rapamycin; SA, streptavidin; TRPML1, transient receptor potential channel mucolipin 1. (PDF) [file pbio.3000252.s003.pdf]

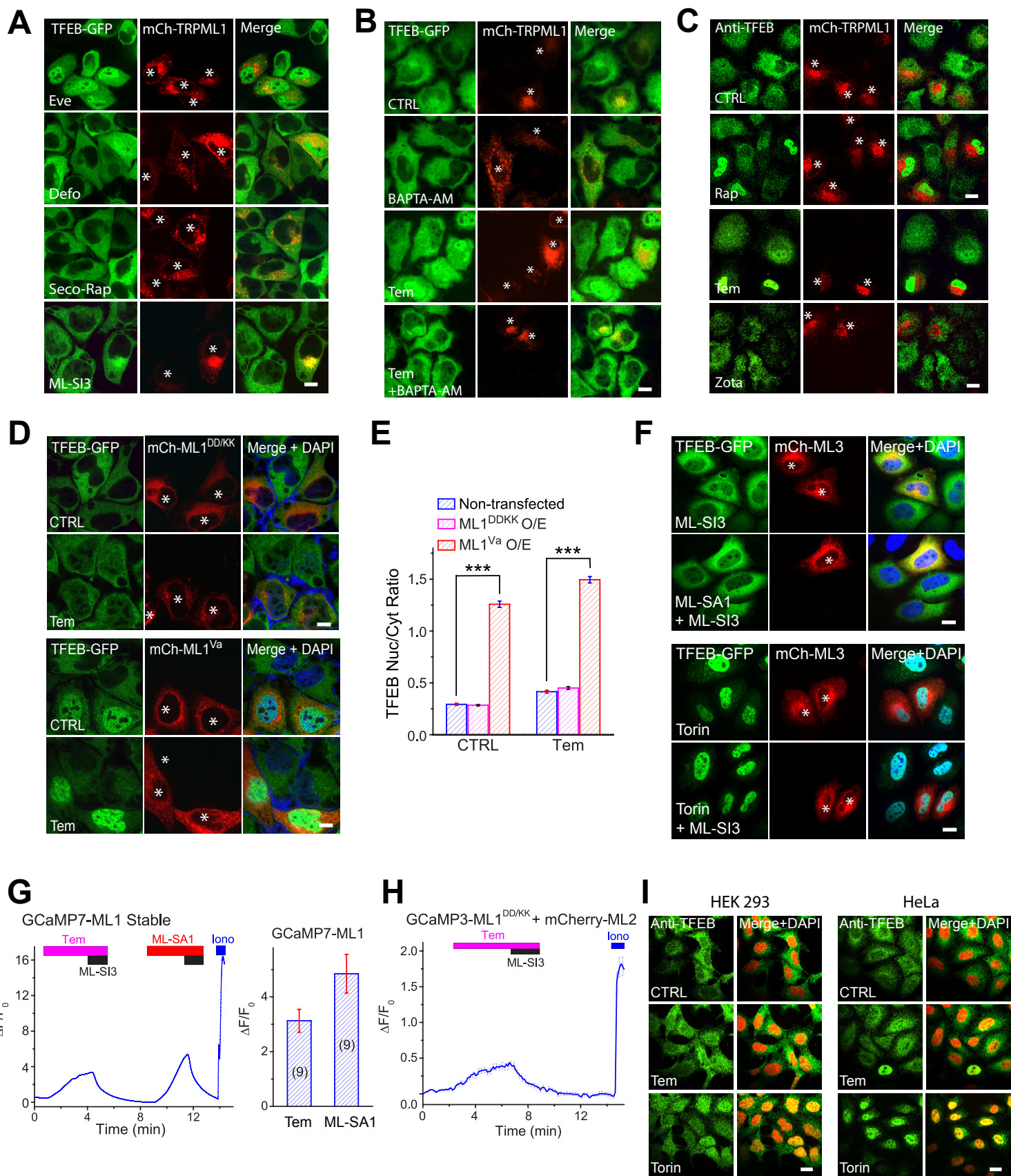

Supplement: S4 Fig — (A) Eve (5 μM, 2 h) induced TFEB nuclear translocation in TFEB-GFP stable cells overexpressing mCherry-TRPML1 (indicated by asterisks). In contrast, no obvious TFEB nuclear translocation was seen with Defo (5 μM, 2 h), Seco-Rap (5 μM), or ML-SI3 (10 μM). Scale bar = 10 μm. (B) BAPTA-AM (5 μM, 1 h pretreatment) blocked Tem-induced TFEB nuclear translocation. Scale bar = 10 μm. (C) Rap (5 μM, 2 h) and Tem (5 μM), but not Zota (5 μM), induced endogenous TFEB nuclear translocation in HeLa cells overexpressing mCherry-TRPML1 (indicated by asterisks). Scale bar = 10 μm. (D) Tem showed no effect on TFEB nuclear translocation in cells transfected with TRPML1DD/KK, a channel-dead pore mutant (upper). Overexpression of constitutively active TRPML1Va mutant resulted in nuclear accumulation of TFEB in the absence of Tem (lower). (E) Quantitation of TFEB nuclear translocation of (D) from 30 to 40 cells in 3 independent experiments. (F) The effects of ML-SI3 (10 μM, 1 h) pretreatment on ML-SA1– and Torin-1–induced TFEB nuclear translocation in TFEB-GFP stable cells that were transfected with mCherry-TRPML3 (indicated by asterisks). (G) Tem increased cytosolic Ca2+ levels through TRPML1 activation. In cells stably expressing GCaMP7-TRPML1, Tem (50 μM) and ML-SA1 (5 μM) increased GCaMP7 fluorescence intensity, which was blocked by ML-SI3 (10 μM) coapplication (left). Iono (1 μM) was used as a positive control. The effects of Tem were quantified from 9 independent experiments (right) and presented as mean ± SEM. (H) The effects of Tem (50 μM) on cytosolic Ca2+ levels in HEK293 cells that were cotransfected with mCherry-TRPML2 and GCaMP3-TRPML1DD/KK. (I) Tem (10 μM, 9 h) failed to induce TFEB (green) nuclear translocation in HEK293 and HeLa cells. Note that Torin-1 (1 μM) induced dramatic TFEB nuclear translocation in HeLa cells but mild TFEB nuclear translocation in HEK293 cells. Nuclei were labelled with DAPI (red, pseudo-color). Scale bar = 10 μm. The individual data underlying (E [file pbio.3000252.s004.pdf]

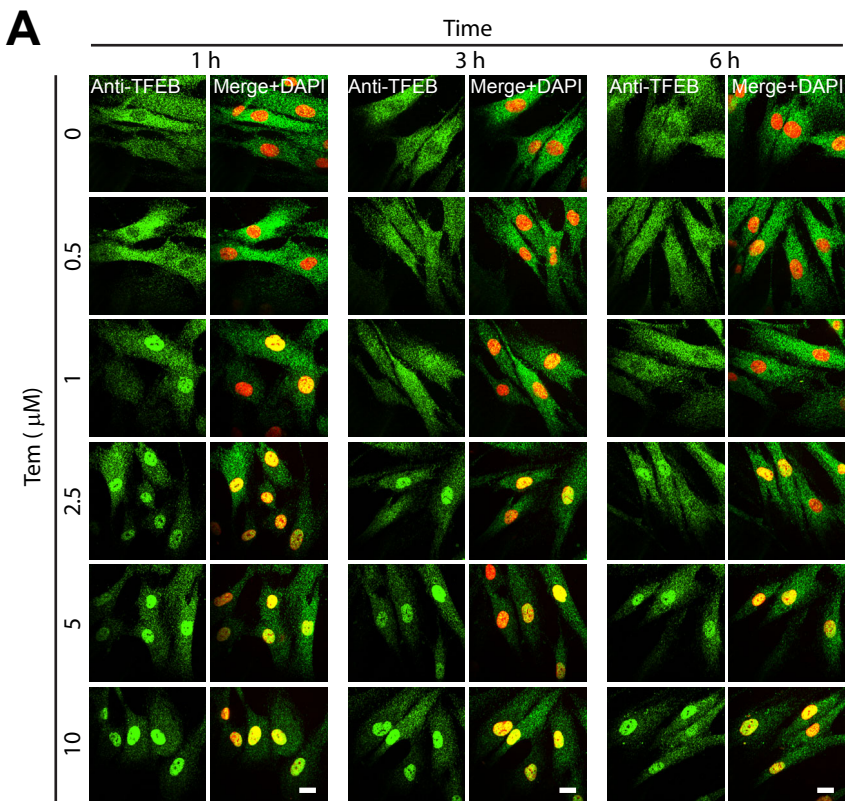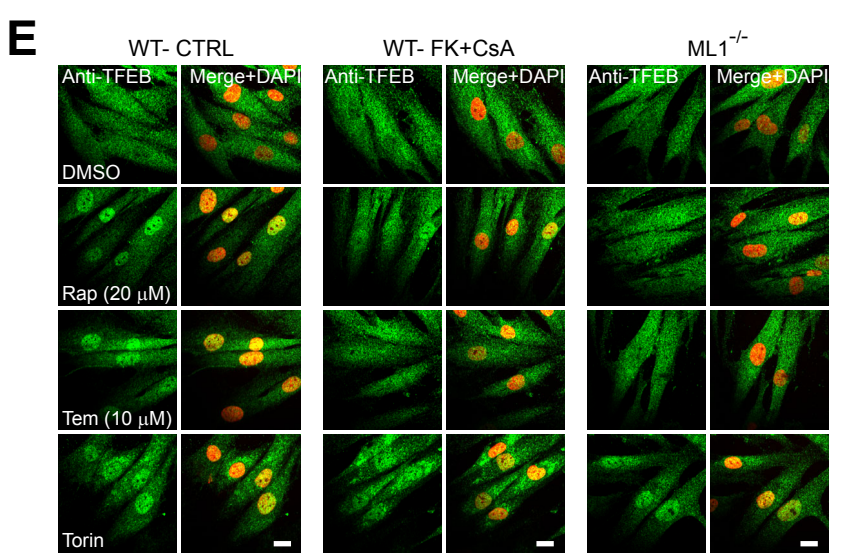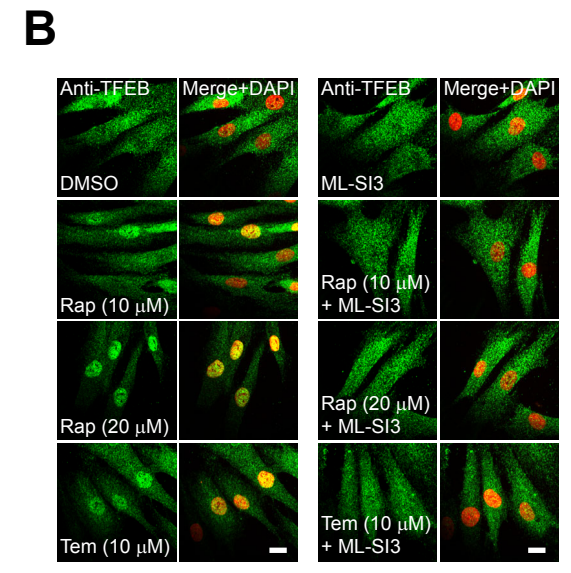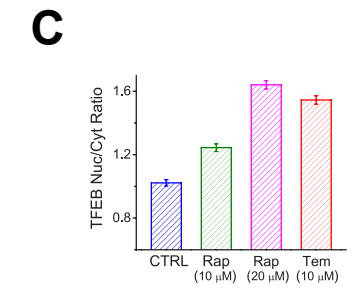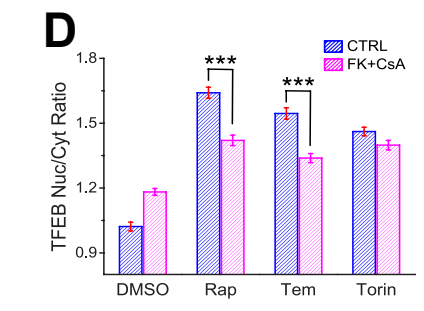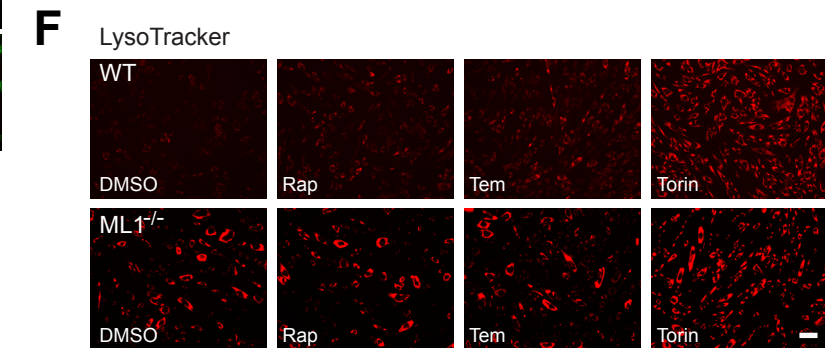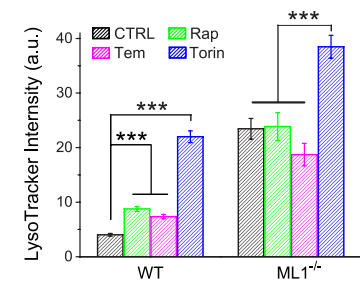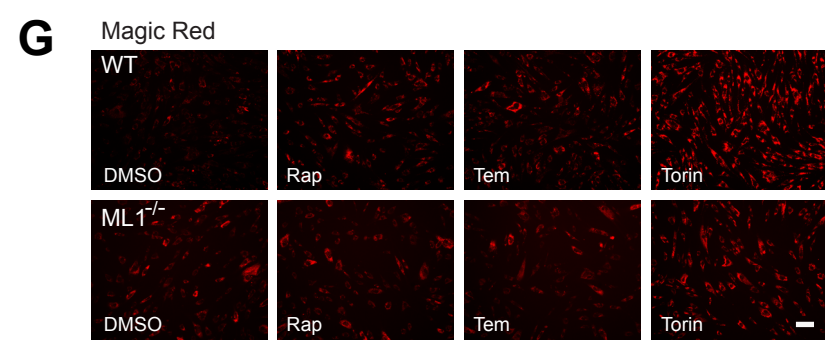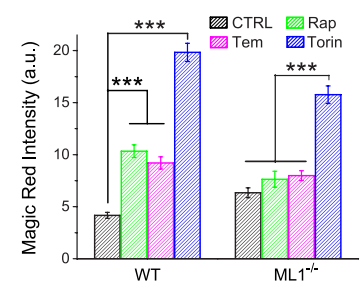

Supplement: S5 Fig — (A) Dose- and time-dependent effects of Tem on TFEB nuclear translocation. Scale bar = 10 μm. (B) Rap and Tem effects on TFEB nuclear translocation in human fibroblasts. Scale bar = 10 μm. (C) Quantification of Rap and Tem effects shown in (B). (D, E) The effects of calcineurin inhibitors FK506 (5 μM) and CsA (10 μM) on Rap- and Tem-induced TFEB nuclear translocation in WT and ML1-/- human fibroblasts. Scale bar = 10 μm. (F) Effects of Rap (20 μM, 6 h) and Tem (10 μM, 6 h) on LysoTracker staining in WT and ML1-/- human fibroblasts. Torin-1 (1 μM, 6 h) was used as a control. Scale bar = 100 μm. (G) The effects of Rap (20 μM, 6 h) and Tem (10 μM, 6 h) on Magic Red staining in WT and ML1-/- cells. Torin-1 (1 μM, 6 h) was used as a control. Scale bar = 100 μm. Averaged data shown in the left panels of (F) and (G) were from 3 independent experiments and are presented as mean ± SEM. ***P < 0.001, one-way ANOVA. The individual data underlying C, D, F, and G can be found in S1 Data. CsA, cyclosporine A; FK506, tacrolimus; ML1−/−, Mucolipidosis IV; Rap, rapamycin; Tem, temsirolimus; TFEB, transcription factor EB; TRPML1, transient receptor potential channel mucolipin 1; WT, wild type. (PDF) [file pbio.3000252.s005.pdf]

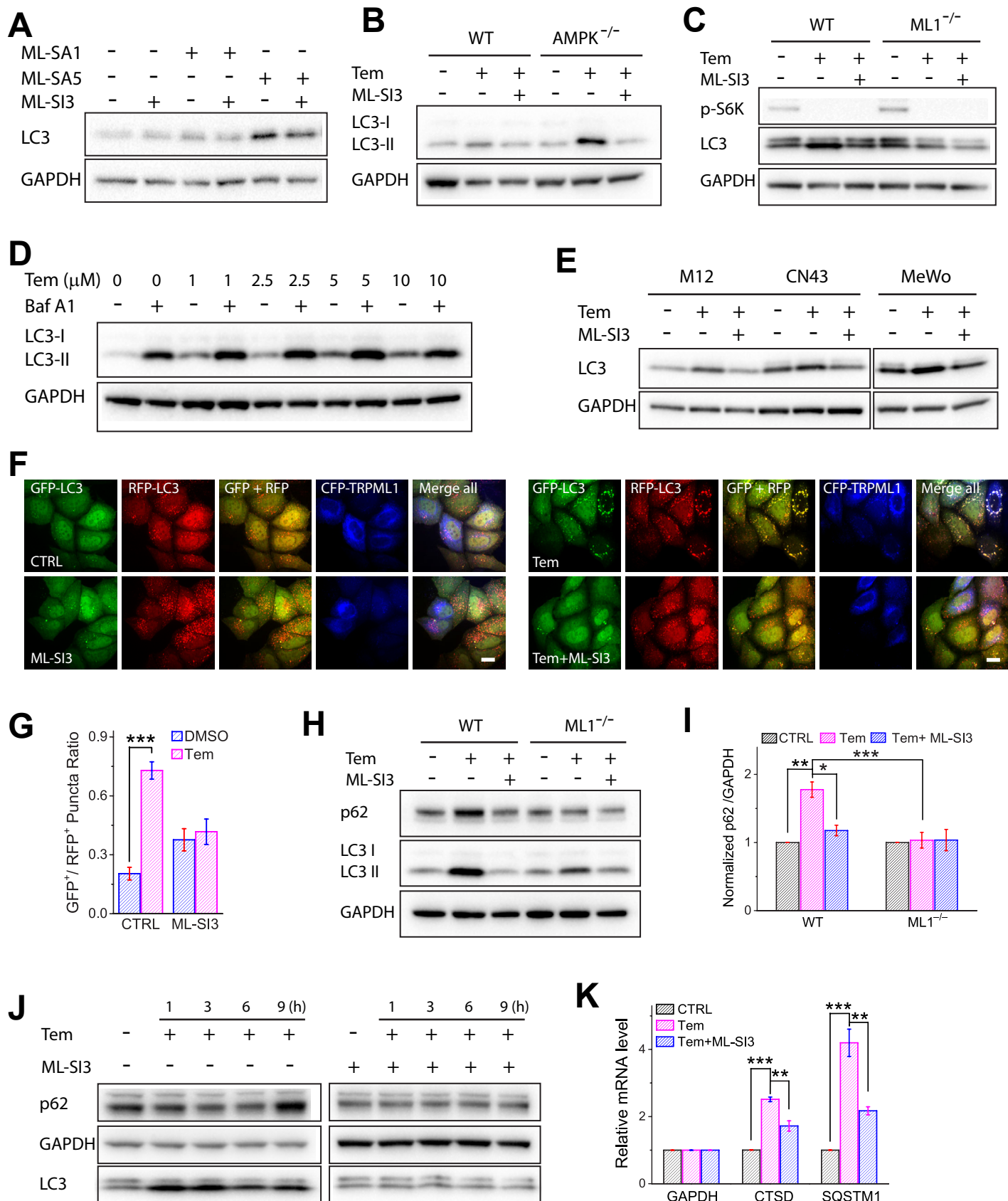

Supplement: S6 Fig — (A) TRPML1 synthetic agonists ML-SA1 (10 μM, 4 h) and ML-SA5 (1 μM, 4 h) increased LC3-II levels in WT human fibroblasts, and the increase was suppressed by ML-SI3 (10 μM). (B) Tem effect in AMPK α1/α2 double KO MEFs. (C) The effects of ML-SI3 (10 μM) on Tem-induced increases in the LC3-II levels and mTOR inhibition in WT human fibroblasts. (D) The effects of Baf-A1 (0.5 μM, 9 h) on Tem-induced LC3-II increases in WT human fibroblasts (left). (E) The effects of TRPML1 inhibitors on the Tem (10 μM, 9 h) in M12 (prostate cancer), CN34 (breast carcinoma), and MeWo (melanoma) cells. (F) Tem (10 μM, 2 h) significantly increased GFP+RFP+ puncta in GFP-RFP-LC3 stable HeLa cells overexpressing CFP-TRPML1. Tem effect was inhibited by ML-SI3 (10 μM). (G) Quantification of F from more than 20 CFP-positive cells for each treatment. (H) Tem (10 μM, 9 h) increased p62 levels in WT but not ML1-/- human fibroblasts. Tem effects in WT cells were blocked by ML-SI3 (10 μM). (I) Quantification of H. (J) Time-dependent effects of Tem (10 μM) on p62 and LC3 protein levels. (K) The effects of Tem (10 μM, 16 h) and ML-SI3 (10 μM) on p62/SQSTM1 transcript levels, analyzed by RT-qPCR. The black framed boxes indicate images coming from separated gel runs. Data shown in G, I, and K were obtained from at least 3 independent experiments and are presented as mean ± SEM. The individual data can be found in S1 Data. *P < 0.05, **P < 0.01, ***P < 0.001, one-way ANOVA. AMPK, 5' adenosine monophosphate-activated protein kinase; Baf-A1, Bafilomycin A1; CFP, Cyan Fluorescent Protein; GFP, green fluorescent protein; HeLa, Henrietta Lacks cells; KO, knockout; LC3-II, microtubule-associated proteins 1A/1B light chain 3B-II; MEF, mouse embryonic fibroblast; ML1−/−, Mucolipidosis IV; ML-SA, TRPML1 synthetic agonist; ML-SI3, TRPML1 synthetic inhibitor 3; mTOR, mechanistic target of rapamycin; p62/SQSTM1, Sequestosome-1; RFP, red fluorescent protein; RT-qPCR, quantitative real-time polymerase chain reaction; T [file pbio.3000252.s006.pdf]

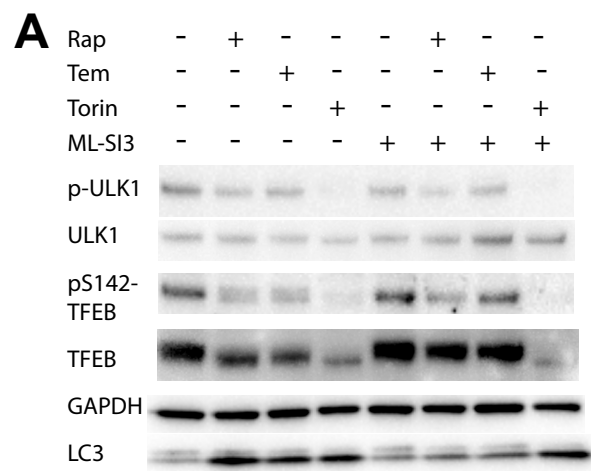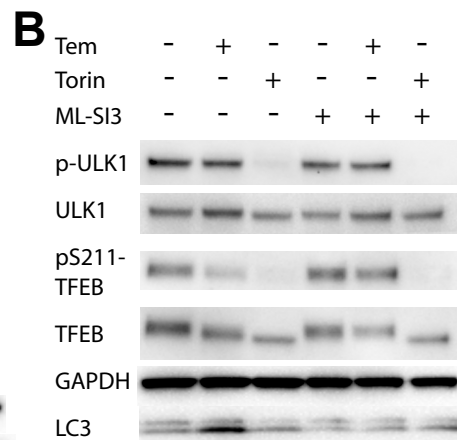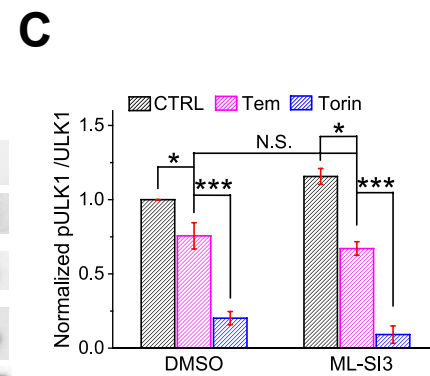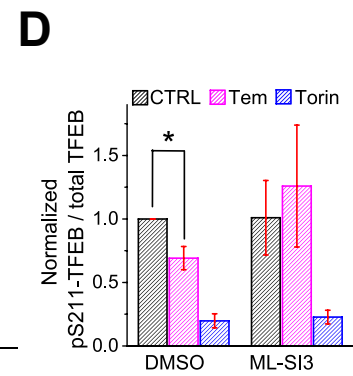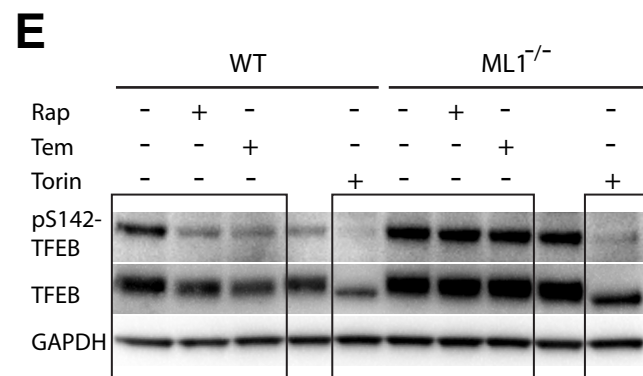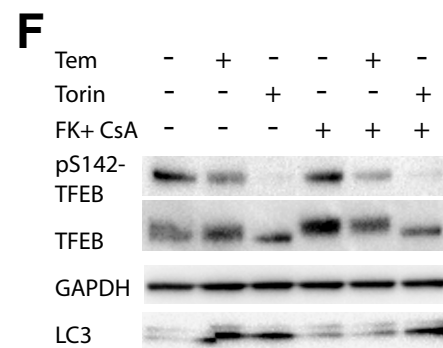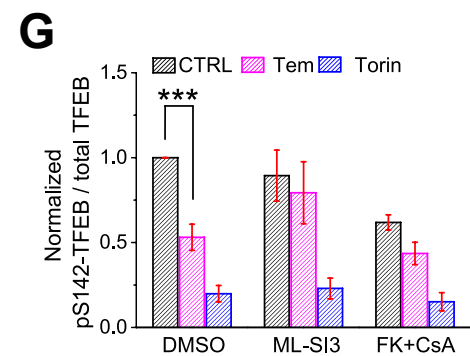

Supplement: S7 Fig — (A) The effects of Rap, Tem, and ML-SI3 on p-ULK1 and pS142-TFEB. (B) The effects of Tem and ML-SI3 on pS211-TFEB. (C) Quantification of p-ULK1. (D) Quantification of Tem effect on pS211-TFEB with or without ML-SI3. (E) The effect of Tem, Rap, and Torin-1 (shown in boxes) on pS142-TFEB in WT and ML1-/- human fibroblasts. (F) The effects of calcineurin inhibitors and Tem on pS142-TFEB. (G) Quantification of pS142-TFEB under various treatment conditions. Data shown in C, D, and G were obtained from at least 3 independent experiments and are presented as mean ± SEM. The individual data can be found in S1 Data. *P < 0.05, **P < 0.01, ***P < 0.001, one-way ANOVA. ML1−/−, Mucolipidosis IV; ML-SI3, TRPML1 synthetic inhibitor 3; N.S., not significant; pS142-TFEB, phospho-TFEB at Ser 142; pS211-TFEB, phospho-TFEB at Ser 211; p-ULK1, phospho-ULK1 at Ser 758; Rap, rapamycin; Tem, temsirolimus; TFEB, transcription factor EB; TRPML1, transient receptor potential channel mucolipin 1; WT, wild type. (PDF) [file pbio.3000252.s007.pdf]
